# Research on the Development of Attitude and Trajectory Measurement Technologies for Oil and Gas Drilling

**DOI:** 10.3390/s26216774

**Published:** 2026-10-26

**Authors:** Shaowen Ji, Longjun Ran, Chunxi Zhang, Dinan Jiang, Xin Lu

**Affiliations:** 1 School of Instrumentation and Optoelectronic of Engineering, Beihang University, Beijing 100191, China; jishaowen93y@buaa.edu.cn (S.J.); ranlongjun@buaa.edu.cn (L.R.); zhangchunxi@buaa.edu.cn (C.Z.); 2 National Key Laboratory of Inertial Measurement, Beijing 100191, China; 3 China Oilfield Services Limited, Langfang 065201, China; jiangdn@cosl.com.cn; 4 School of Transportation Science and Engineering, Beihang University, Beijing 100191, China

**Keywords:** wellbore attitude, wellbore trajectory, inertial navigation, magnetometer, gyroscope

## Abstract

Wellbore attitude and trajectory are critical parameters for complex oil and gas drilling and geosteering. As global energy exploration extends to deep-earth, deep-water, and unconventional resources, higher demands are imposed on downhole navigation accuracy and real-time performance. This paper reviews the development status and future trends of wellbore attitude and trajectory measurement technologies. Attitude measurement mechanisms based on magnetometers and gyroscopes are comparatively analyzed, along with their respective research focuses. Recent advances in magnetic interference compensation and accuracy enhancement for magnetometer-based schemes are summarized. Differences in attitude determination algorithms between reduced and full gyroscope configurations are compared, and the common issue of performance degradation in near-vertical sections due to reduced accelerometer SNR is examined via numerical simulation. For trajectory measurement, traditional discrete reconstruction methods and their station-interval-induced accuracy limitations are evaluated, highlighting the urgent need for a transition from static discrete to dynamic continuous measurement. Strapdown inertial navigation-based real-time estimation methods are discussed, with error propagation characteristics analyzed. Kinematic constraints such as depth increments and zero-velocity updates are identified as effective means to mitigate inertial error divergence. Finally, future prospects are outlined, emphasizing that developing an integrated “underground positioning system” combining quantum sensing, multi-sensor fusion, and AI-assisted data processing represents an important direction for advancing intelligent drilling measurement and control.

## 1. Introduction

Wellbore attitude and trajectory are critical parameters for decision-making in directional drilling and geosteering. As global energy demand shifts toward deep-earth, deep-water, and unconventional oil and gas resources, cluster wells, extended-reach wells, and deep wells have gradually become common well types in oil and gas resource development. These complex well types impose higher requirements on wellbore attitude measurement and trajectory control. Wellbore attitude and trajectory measurement runs through all stages of oil and gas well development. For example, in the wellpath design stage, wellbore attitude serves as the fundamental parameter for trajectory design. During the drilling operation phase, in measurement, trajectory correction, and anti-collision operations, wellbore attitude parameters are typically transmitted uphole to the surface system via downhole communication systems. The surface software then analyzes drilling quality, evaluates drilling safety, and compares the deviations from the preset trajectory [1]. In the casing and completion phase, a follow-up survey of the wellbore orientation is usually conducted to obtain more precise positional parameters [2].

**Figure 1:**
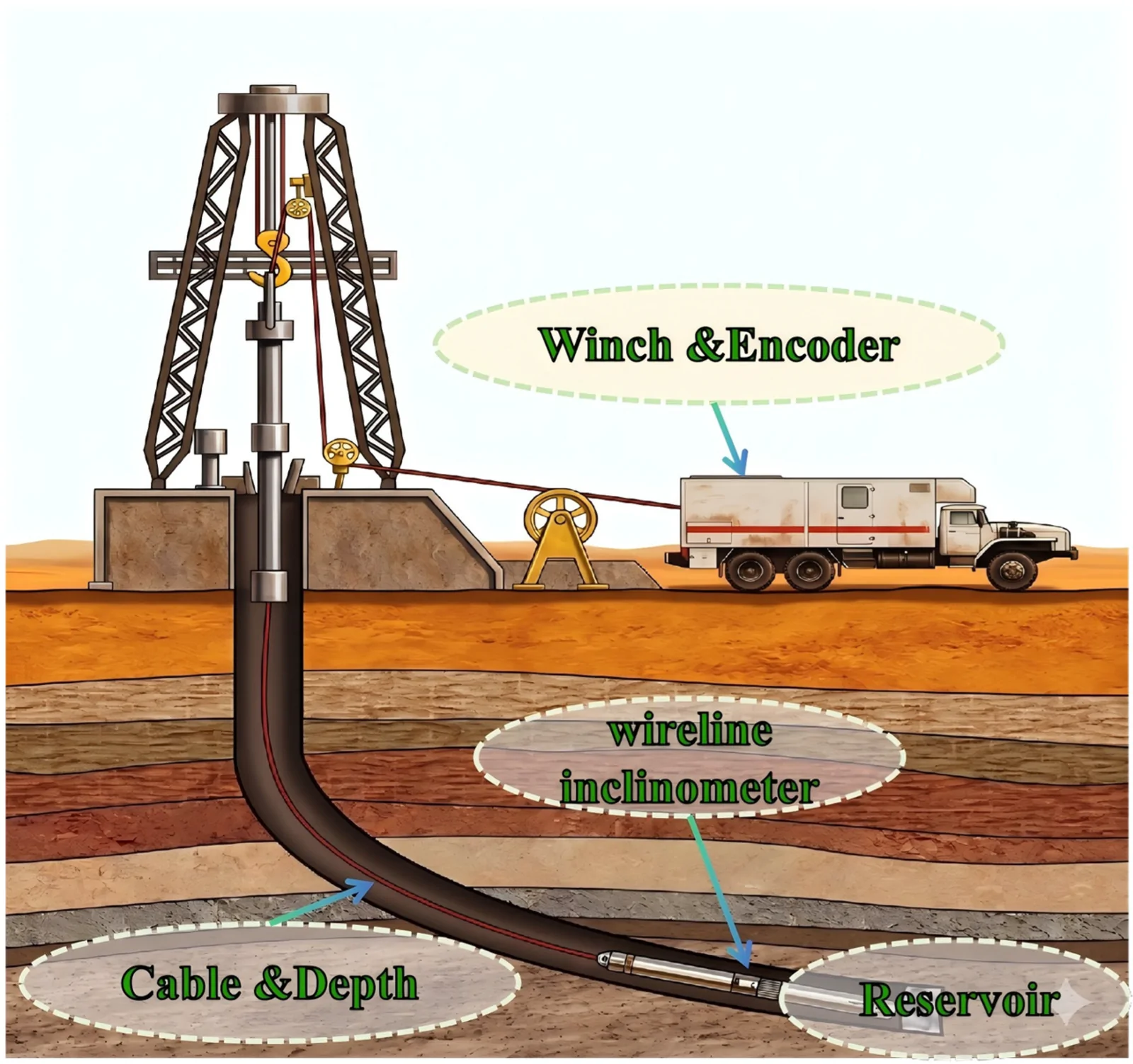
Operating Scenario of the Wired Inclinometer.

Two types of instruments are commonly employed for wellbore attitude measurement during oil and gas well development: wireline cable inclinometers and wireless measurement while drilling (MWD) systems. The operating principle of the wireline cable inclinometer is schematically depicted in Figure 1. A surface winch lowers the inclinometer into the wellbore via a cable, and during the running-in process, the inclinometer continuously records wellbore attitude parameters, including inclination angle, azimuth angle, and toolface angle. Simultaneously, the depth is registered in real time by the reel mounted on the winch. By establishing a spatiotemporal alignment between the depth readings and the attitude measurements, the wellbore trajectory can be reconstructed and plotted.

**Figure 2:**
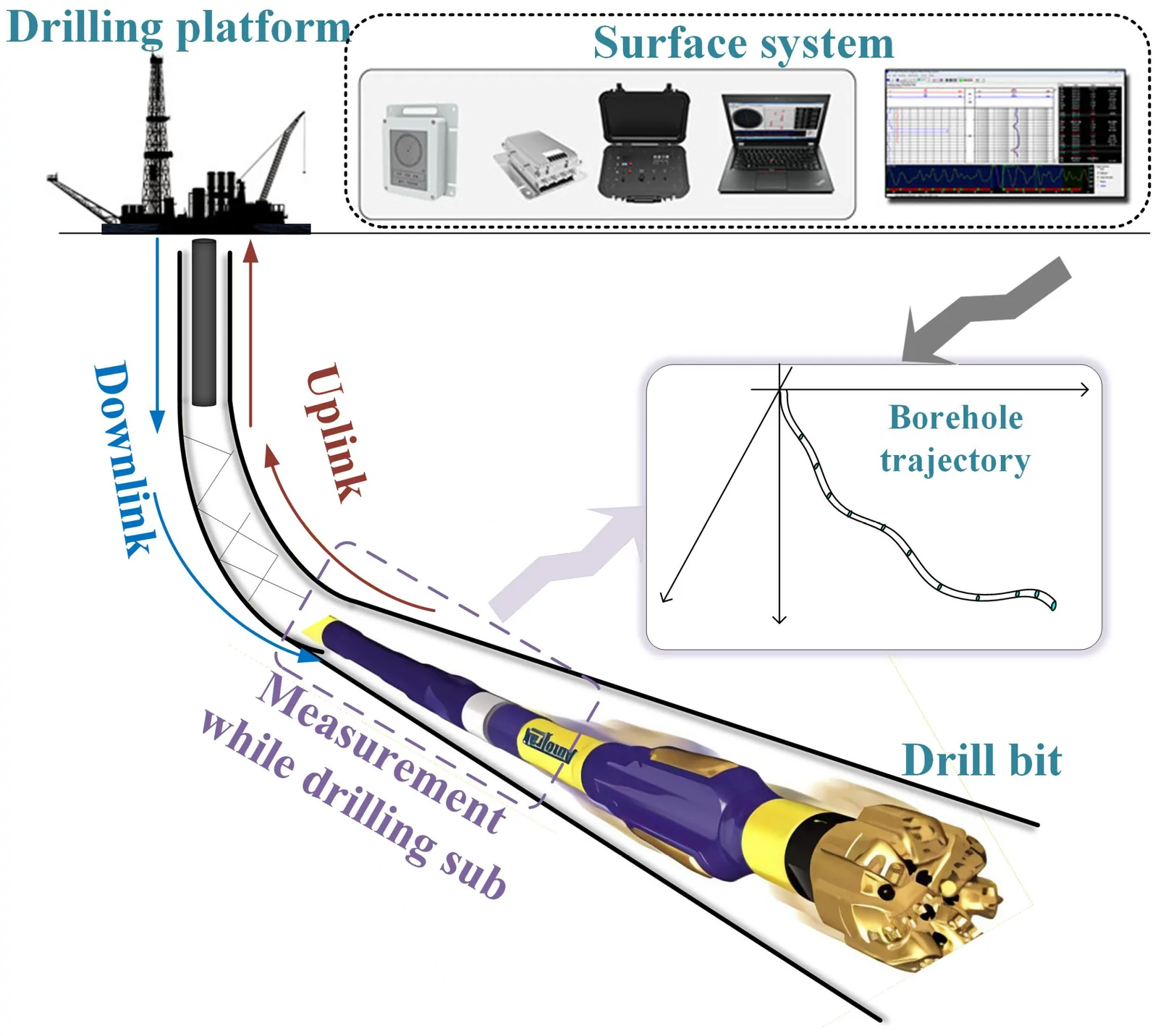
Operating scenario of the wireless MWD sub.

Wireless MWD systems measure wellbore attitude during drilling operations through wireless telemetry methods such as mud pulse transmission [3]. As illustrated in Figure 2, the MWD sub is positioned within the drill collar immediately behind the drill bit and, together with the communication module, forms the MWD system. When a certain depth interval has been drilled and a survey station is reached, a command is issued from the surface system, and the drilling process is typically paused for several minutes to conduct azimuth measurements. Upon completion of the measurement, the mud pulse telemetry module is activated to transmit the wellbore attitude data to the surface software platform [4]. The wellbore trajectory is reconstructed by the surface software through the integration of wellbore attitude measurements at each survey station and the corresponding well depths between adjacent stations, with the latter being predominantly governed by the drill pipe length.

Two types of sensor configurations are commonly employed for wellbore attitude measurement: one utilizing a magnetometer combined with accelerometers, and the other employing a gyroscope in conjunction with accelerometers. In the former configuration, the reference for azimuth measurement is the magnetic north, whereas in the latter, the reference is the geographic north. The magnetometer is susceptible to magnetic interference and therefore requires periodic compensation and calibration. When hard-iron or soft-iron magnetic disturbances are present in the wellbore measurement environment, the magnetometer becomes inoperative, and the gyroscope must be relied upon to perform azimuth measurements [5]. The gyroscopes applied in oil and gas well development can be further categorized into mechanical gyroscopes, Micro-Electro-Mechanical System (MEMS) gyroscopes, and fiber optic gyroscopes. Gyroscopes with solid-state packaging characteristics, such as optical gyroscopes, are better suited to withstand the downhole mechanical environment [6]. However, they face challenges in adapting to high-temperature or ultra-high-temperature operating conditions. Table 1 summarizes the typical wellbore attitude measurement accuracies achieved under various sensor configuration schemes. The azimuth accuracy and toolface accuracy specified in the table are generally constrained to conditions where the inclination angle is greater than 3°, because in near-vertical states (inclination angle less than 3°), the signal-to-noise ratio of the radial accelerometers degrades significantly, leading to a substantial reduction in accuracy. In near-vertical well sections, a magnetic toolface or gyro toolface is typically used for orientation purposes. This issue will be further analyzed in the following sections.

Wellbore attitude angles are critical parameters for drill bit trajectory control and wellbore trajectory measurement. The depth intervals between successive survey stations recorded during the drilling process, when combined with azimuth and inclination angles, enable the determination of positional offsets in each direction [9]. The accuracy of trajectory measurement is influenced not only by the precision of attitude angles but also by systematic drilling errors and environmental disturbances [10]. Since the propagation mechanisms of various error sources cannot be accurately described mathematically during the drilling process, wellbore trajectory uncertainty analysis theory is introduced to constrain the error bounds of the trajectory [11]. In technical workflows such as wellpath design, wellbore trajectory measurement, and wellbore trajectory prediction, the trajectory error ranges in all directions are taken into consideration to mitigate operational risks such as the inability of the drilling assembly to reach the target reservoir [12].

This paper presents a comprehensive review of the current state of wellbore attitude and trajectory measurement technologies in the field of oil and gas drilling. Proceeding from the measurement principles, the technical characteristics and research directions of wellbore attitude measurement methods based on different sensor configurations are discussed. In conjunction with the engineering application characteristics of wellbore trajectory measurement technologies, the theoretical framework and development trends of trajectory measurement in oil and gas drilling are systematically elaborated. The remainder of this paper is organized as follows. Section 2 introduces the principles of wellbore attitude measurement and discusses the current research status, as well as the advantages and disadvantages, of two types of sensors—magnetometers and gyroscopes—in wellbore attitude measurement. Section 3 presents the measurement principles and technical features of conventional discrete trajectory reconstruction methods and analyzes the feasibility of continuous wellbore trajectory measurement. Section 4 provides an outlook on future development directions of wellbore attitude and trajectory measurement technologies. Section 5 concludes this paper.

## 2. Wellbore Attitude Measurement Technology

### 2.1. Definition of Coordinate Systems and Attitude Angles

To ensure consistency in attitude representation, the coordinate systems and attitude angles used in this study are defined. Following the conventions of inertial navigation, the navigation coordinate system (hereinafter referred to as the n-frame), also known as the geographic coordinate system, is selected as the reference frame. A body coordinate system (hereinafter referred to as the b-frame) rigidly fixed to the drilling tool is established to describe its spatial attitude. The X*_n_*-, Y*_n_*-, and Z*_n_*-axes of the navigation frame point eastward, northward, and upward, respectively. In the b-frame, the Y*_b_*-axis is aligned with the drilling direction, while the X*_b_*- and Z*_b_*-axes are perpendicular to the tool axis. Together, these three axes form a right-handed coordinate system.

**Figure 3:**
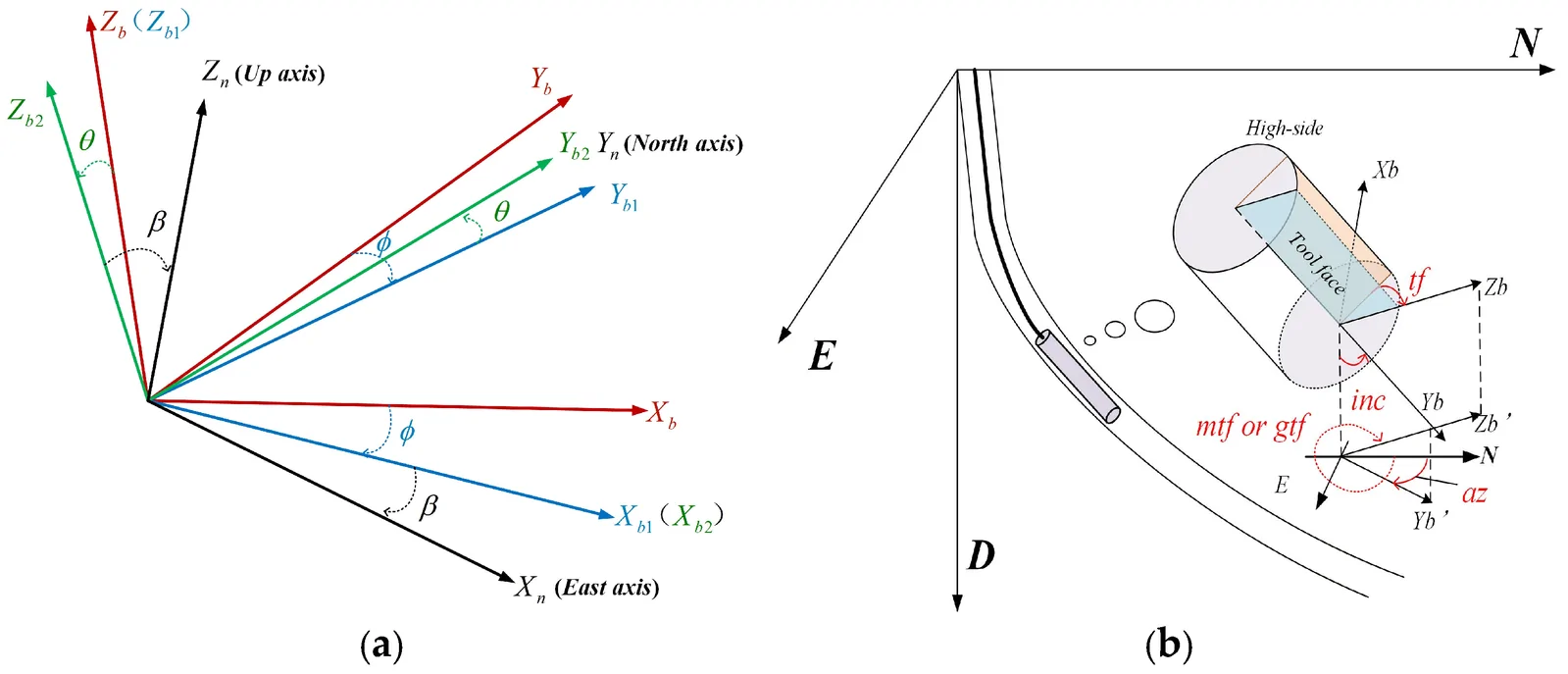
Spatial representation of wellbore attitude: (**a**) definitions of the coordinate systems and Euler-angle rotations; (**b**) spatial representation of the wellbore attitude angles.

As illustrated in Figure 3a, the attitude relationship between the navigation and body frames can be described using Euler-angle rotations. Following the prescribed rotation sequence, i.e., *R_z_*(−*ϕ*)→*R_x_*(*θ*)→*R_y_*(*β*), the b-frame can be rotated into alignment with the navigation frame. Here, *ϕ* denotes the heading or azimuth angle, which is measured clockwise from geographic north toward east and ranges from 0° to 360°. The angle θ denotes the pitch angle and ranges from −90° to 90°. It is defined as 0° when the Y*_b_*-axis is horizontal, −90° when the Y*_b_*-axis points vertically downward, and 90° when it points vertically upward. The angle *β* denotes the roll angle, which ranges from 0° to 360°, with counterclockwise rotation about the Y*_b_*-axis defined as positive. Based on these definitions, the coordinate transformation matrix can be expressed as Equation (1). Because this matrix is parameterized by the three attitude angles, it is also referred to as the attitude matrix.

(1) $$C_{b}^{n}=\left(\begin{array}{ccc} \cos\;\phi \cos\;\beta +\sin\;\phi \sin\;\theta \sin\;\beta & -\sin\;\phi \cos\;\theta & \cos\;\phi \sin\;\beta -\sin\;\phi \sin\;\theta \cos\;\beta \\ -\sin\;\phi \cos\;\beta +\cos\;\phi \sin\;\theta \sin\;\beta & \cos\;\phi \cos\;\theta & -\sin\;\phi \sin\;\beta -\cos\;\phi \sin\;\theta \cos\;\beta \\ -\cos\;\theta \sin\;\beta & \sin\;\theta & \cos\;\theta \cos\;\beta \end{array}\right)$$

The wellbore attitude is characterized by the azimuth angle (*az*), inclination angle (*inc*), and gravity toolface angle (*tf*). The gravity toolface angle is defined consistently with the roll angle and represents the angle between the high side of the drilling tool and the sensitive axis of a designated radial accelerometer. In near-vertical conditions, the angle between the reference line and the magnetic north (or true north) is used as an approximation, which is termed magnetic toolface (*mtf*) or gyro toolface (*gtf*). The inclination angle, *inc*, theoretically ranges from 0° to 180° and is related to the pitch angle, *θ*, by *inc* = 90° + *θ*. When the tool axis points vertically downward, *inc* = 0°; when the tool axis is horizontal, *inc* = 90°. In conventional drilling operations, the tool generally advances downward or in a near-horizontal direction, whereas upward-drilling sections are relatively uncommon. Consequently, the inclination angle predominantly falls within the range of [0°, 90°] and enters the range of (90°, 180°] only in upward-curving well sections or under specific trajectory designs. A spatial representation of the wellbore attitude angles is shown in Figure 3b. The inclination and azimuth angles are the two essential parameters used for wellbore trajectory reconstruction. According to the definitions of the wellbore attitude angles, the transformation matrix $C_{b}^{n}$ can be reformulated as follows:

(2) $$C_{b}^{n}=\left(\begin{array}{ccc} \cos\;\mathrm{az}\cos\mathrm{tf}-\sin\;\mathrm{az}\cos\;\mathrm{inc}\sin\;\mathrm{tf} & \sin\;\mathrm{azsin}\;\mathrm{inc} & \cos\;\mathrm{az}\sin\;\mathrm{tf}+\sin\;\mathrm{azcos}\;\mathrm{inc}\cos\;\mathrm{tf}\\ -\sin\;\mathrm{az}\cos\;\mathrm{tf}-\cos\;\mathrm{azcos}\;\mathrm{inc}\sin\;\mathrm{tf} & \cos\;\mathrm{azsin}\;\mathrm{inc} & -\sin\;\mathrm{az}\sin\;\mathrm{tf}+\cos\;\mathrm{az}\cos\;\mathrm{inc}\cos\;\mathrm{tf}\\ -\sin\;\mathrm{inc}\sin\;\mathrm{tf} & -\cos\;\mathrm{inc} & \sin\;\mathrm{inc}\cos\;\mathrm{tf} \end{array}\right).$$

### 2.2. Magnetometer-Based Wellbore Attitude Measurement Method

The magnetometer, which is sensitive to the strength and direction of the Earth’s weak magnetic field, represents the earliest vector sensor applied to wellbore attitude measurement. The magnetometer probe features a robust physical structure capable of withstanding vibration and shock loads encountered during drilling operations. In addition, the magnetometer exhibits low power consumption, making it suitable for prolonged operation within the confined downhole space. In a magnetically undisturbed environment, it can be combined with accelerometers to achieve high-precision azimuth measurements. The governing equations for wellbore attitude calculation are presented as

(3) $$\{\begin{array}{c} \mathrm{inc}=\arcsin\left(\frac{\sqrt{{g_{x}}^{2}+{g_{z}}^{2}}}{g}\right)\\ \mathrm{tf}=\arctan\left(\frac{-g_{x}}{g_{z}}\right)\\ \mathrm{az}=\arctan\left(\frac{m_{x}\mathrm{costf}+m_{z}\mathrm{sintf}}{\left(m_{x}\mathrm{sintf}-m_{z}\mathrm{costf}\right)\mathrm{sininc}+m_{y}\mathrm{cosinc}}\right) \end{array},$$

where *g_x_* and *g_z_* denote the accelerometer outputs along the *x*-axis and *z*-axis, respectively; *g* is the gravitational acceleration constant; and *m_x_*, *m_y_* and *m_z_* represent the magnetometer outputs along each axis. It can be observed from the above equations that *inc* and *tf* are primarily determined by the three-axis accelerometer. In inertial navigation terminology, these two angles are also referred to as horizontal angles, corresponding to the pitch and roll angles of an aircraft [13]. Only after the navigation horizontal plane is established by the carrier can the projection of the flight direction onto the horizontal plane be accurately computed, thereby enabling the determination of the angle relative to true north or magnetic north. During drilling or logging operations, *mtf* or *gtf* is typically utilized as a substitute for gravity toolface when the instrument is in a near-vertical attitude. This is because, under near-vertical conditions, the radial accelerometer components used for toolface determination approach “0 g,” resulting in accuracy degradation, which in turn adversely affects the azimuth calculation.

Ideally, the measurement accuracy of magnetometer azimuth is far higher than practical engineering requirements. However, significant soft-iron or hard-iron interference usually exists in downhole environments. For example, ferromagnetic materials from casings in shallow or adjacent wellbores can introduce additional interference magnetic fields; meanwhile, drill string materials, downhole circuit systems, and metallic minerals in formations during drilling can also generate time-varying interference magnetic fields [14]. Therefore, research on magnetometer-based wellbore attitude measurement technology mostly focuses on the compensation and calibration of magnetometers to improve azimuth measurement accuracy [15,16]. Hard-iron interference manifests as a fixed offset superimposed on each axis output, whereas soft-iron interference affects the scale factor and orthogonality of the magnetometer. The general form of the magnetometer error model is shown in Equation (4).

(4) $$M^{b}=\left[\begin{array}{ccc} u_{\mathrm{xx}} & u_{\mathrm{xy}} & u_{\mathrm{xz}}\\ u_{\mathrm{yx}} & u_{\mathrm{yy}} & u_{\mathrm{yz}}\\ u_{\mathrm{zx}} & u_{\mathrm{zy}} & u_{\mathrm{zz}} \end{array}\right]\left[\begin{array}{c} M_{x}^{s}\\ M_{y}^{s}\\ M_{z}^{s} \end{array}\right]+\left[\begin{array}{c} v_{\mathrm{mx}}\\ v_{\mathrm{my}}\\ v_{\mathrm{mz}} \end{array}\right]=U_{m}M^{s}+V_{m}$$

In Equation (4), ***M***^s^ represents the actual output of each axis sensor, with the superscript s denoting the sensor coordinate frame; ***M****^b^* represents the accurate value after error compensation, with the superscript b denoting the body coordinate frame used for subsequent attitude navigation. The diagonal elements of matrix ***U****_m_* correspond to the scale factor errors induced by soft-iron effects, while the non-diagonal elements represent the non-orthogonality errors caused by soft-iron interference. ***V****_m_* denotes the zero-bias error resulting from hard-iron interference. Existing research efforts on magnetometer error compensation methods are built upon this error model.

To ensure accurate error compensation for the magnetometer, a common traditional method involves fixing the instrument on a non-magnetic three-axis turntable [17,18]. By sampling the sensor outputs across multiple orientations and combining them with methods like least squares or polynomial fitting, all 12 error terms in the equation can be solved. Although this type of method demands strict experimental environments and is time-consuming, it can accurately compensate for each individual error term and remains widely adopted in current engineering applications.

Another class of methods involves capturing sensor outputs in all directions by rotating the magnetometer, from which an ellipsoid equation containing error coefficients is derived based on the general magnetometer error model [19,20]. However, the general expression of an ellipsoid contains only 9 coefficients. To address this issue, the matrix is constrained to an upper or lower triangular form, reducing the soft-iron error parameters to 6. This approach is a widely used online error calibration technique known as ellipsoid fitting (EF)—a scalar calibration technique based on the principle of magnitude invariance of the magnetic field vector. The general form of the triaxial magnetometer ellipsoid can be expressed as:

(5) $$\{\begin{array}{l} {M^{s}}^{T}{\mathrm{RM}}^{s}+bM^{s}=1\\ a_{1}{m_{x}}^{2}+a_{2}{m_{y}}^{2}+a_{3}{m_{z}}^{2}+a_{4}m_{x}m_{y}+a_{5}m_{y}m_{z}+a_{6}m_{x}m_{z}+a_{7}m_{x}+a_{8}m_{y}+a_{9}m_{z}=1 \end{array}$$

In the above equation, the matrix ***R*** containing non-orthogonality and scale factor error terms, as well as the matrix ***b*** representing the bias parameters for each axis, can be solved from the coefficients *a*_1_∼*a*_9_. The core of this mathematical-analytical error compensation method lies in establishing an accurate error model and acquiring sufficient sensor data in all directions to enable a more precise estimation of the error parameters.

**Figure 4:**
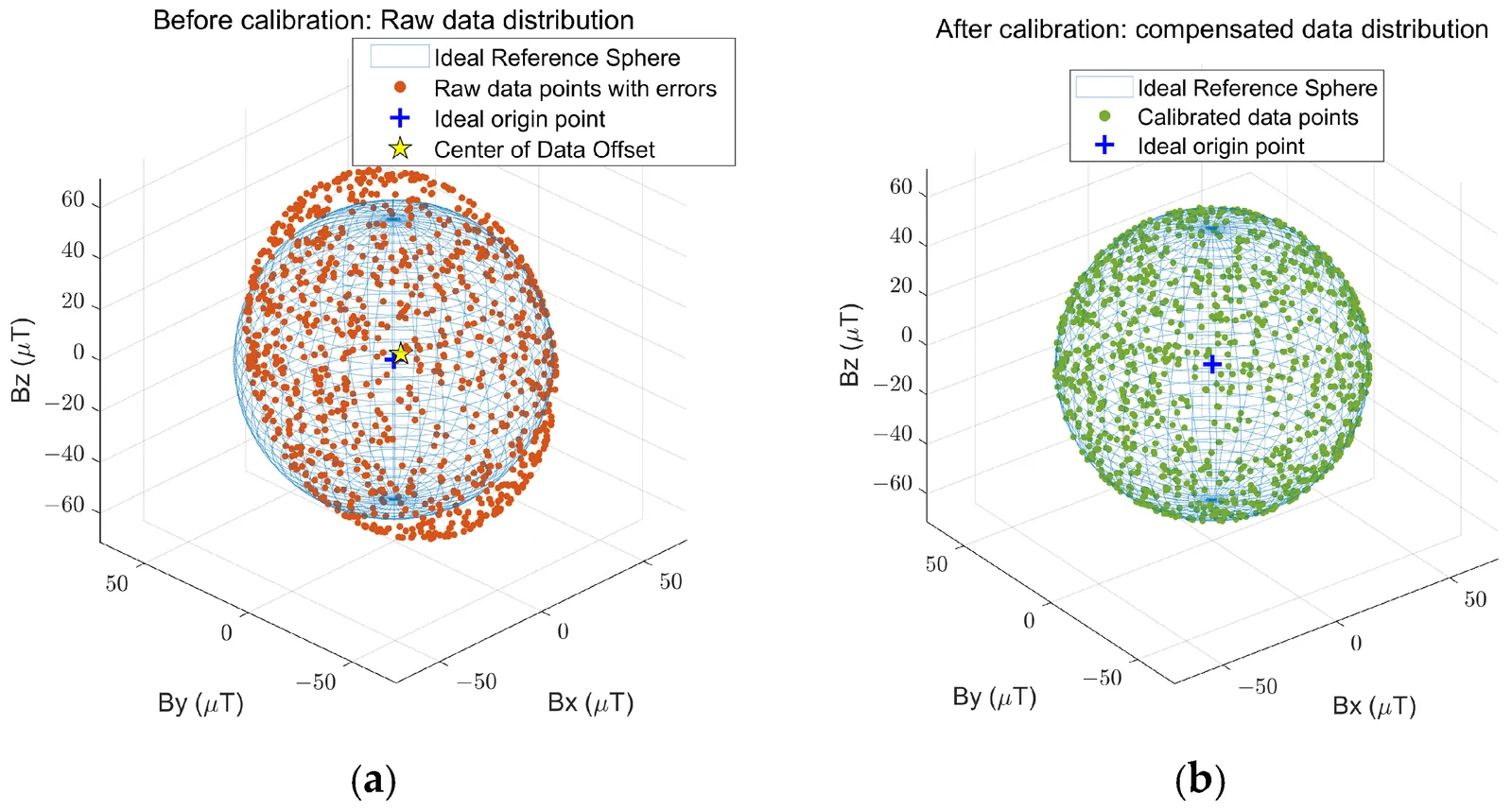
Comparison of triaxial magnetometer before and after compensation based on EF method. (**a**) Spatial distribution of triaxial magnetometer data with errors. (**b**) Spatial distribution of triaxial magnetometer data calibrated via ellipsoid fitting.

Figure 4 illustrates a comparison of the data distribution of the triaxial magnetometer before and after error compensation using the ellipsoid fitting method. Non-orthogonality errors in the triaxial magnetometer induce distortion in the generated 3D ellipsoid; scale factor errors cause the data range along a specific axis to deviate from the ideal value; and zero-bias errors shift the center of the ellipsoid away from the coordinate origin. During the fitting process, the raw data should be uniformly distributed across all spatial directions to ensure that the least squares method can estimate the error parameters with higher accuracy.

Addressing ellipsoid fitting and static multi-position calibration techniques, numerous researchers have devoted efforts to algorithmic and theoretical innovations aimed at estimating triaxial magnetometer errors more accurately, thereby enhancing orientation measurement precision. Accelerometers integrated within inclinometers or MWD systems serve as effective auxiliary calibration tools. Given the spatial vector invariance between the gravity field and geomagnetic field measured by accelerometers and magnetometers, respectively, an error model based on the vector product between these two sensors can be established. This model, combined with multi-position static data sampling, enables the estimation of magnetometer errors [21]. Leveraging the invariant angle between the local gravity vector and the magnetic field vector, Yu, H. et al. proposed a novel in-field calibration method called “Inclination-based Calibration” (I-Calibration) [22]. This method reliably estimates magnetometer model parameters using solely a linear least-squares estimator. In this workflow, accelerometer errors are calibrated first; subsequently, in a magnetically clean environment, the calibrated accelerometer acts as a reference baseline to solve for error parameters using numerical methods such as Newton–Raphson iteration or least-squares estimation. Considering downhole operating temperature ranges, Hu et al. established individual sensor and thermal error models and proposed a calibration methodology based on a full-field calibration algorithm, least-squares estimation, and a transfer-matrix model [23]. This approach computes calibration parameters at ambient temperature efficiently and provides an algorithm for deriving temperature coefficients over the target range. For inclinometers composed of gyroscopes, accelerometers, and magnetometers, magnetometer calibration can also leverage the invariants among these three vector fields—provided the gyroscope accurately senses Earth’s rotation rate. For instance, Ji S. et al. utilize Fiber Optic Gyroscopes (FOGs) to assist in calibrating magnetometers. Because magnetized magnetometers exhibit significant errors, leveraging the calibration-free nature of FOGs serves as a viable pathway for online/in-situ calibration [24]. Furthermore, to enable real-time error compensation during operations, Multi-Station Analysis (MSA) has emerged as a typical method in drilling and logging applications. MSA combines sensor data from multiple downhole static survey stations for online error correction, thereby improving wellbore azimuth measurement accuracy [25]. Beyond magnetometer calibration, MSA is also widely applied in evaluating wellbore trajectory uncertainties and optimizing well path placement [26,27].

During drilling and logging operations, errors introduced by environmental noise similarly restrict the accuracy of wellbore attitude measurements. To address this issue, a common strategy involves sensor noise reduction techniques to diminish the magnitude of attitude error. Combining radial accelerometers at the bit end with an axial MEMS gyroscope, Wang W. et al. adopted a nonlinear Complementary Filter (CF) to suppress vibration acceleration impacts, thereby enhancing toolface angle accuracy during dynamic drilling [28]. To improve the precision of inclination measurements in dynamic environments, Yang et al. utilized the periodic characteristics of magnetometer signals—which remain unaffected by shock and vibration—and proposed an Unscented Kalman Filter (UKF) algorithm tailored for mitigating colored noise in accelerometer outputs [29]. Recognizing that geomagnetic component measurements directly affect downhole tool azimuth and magnetic toolface angles, Li et al. proposed a Zonotopic Kalman Filter (ZKF)-based state estimation framework for geomagnetic components and attitude determination [30]. By incorporating the rotational constraint characteristics of Rotary Steerable Systems (RSS), Xue et al. constructed a novel state-space model that leverages the periodic rotation of magnetometers and accelerometers as measurement updates. This KF-based method achieves continuous azimuth measurement, simultaneously boosting wellbore attitude accuracy and effectively dampening drill-string-induced errors [31]. Furthermore, Liu et al. employed an optics clustering algorithm to filter out noise spikes in magnetometer and accelerometer readings, subsequently combining an improved Multi-Station Analysis (MSA) with a Gaussian Process Regression (GPR) model to suppress cross-axis magnetic interference and nonlinear noise [32]. This three-stage strategy effectively enhances magnetic azimuth accuracy under complex magnetic environments.

The evolution of artificial intelligence (AI) algorithms has significantly driven their applications in oil and gas well engineering. To enhance wellbore attitude precision, researchers have conducted extensive explorations centered on AI methodologies. Addressing challenges in drilling trajectory control caused by downhole attitude data loss and sensor noise, Liu et al. proposed a Long Short-Term Memory (LSTM) network model that successfully accomplishes data completion across acceleration and magnetic flux signals, thereby enabling robust sensor data reconstruction [33]. Integrating downhole tool attitude sensor data with machine learning techniques, Liu et al. performed signal inversion on accelerometer readings specifically for dynamic inclination measurement; by training on signal features across varying rotational speeds, the method achieved marked accuracy improvements in dynamic inclination under multi-speed operating conditions [34]. Combining empirical calibration data with measurement error characteristics, Yang et al. established a neural network model based on the K-proximity method to compensate for error components in MWD magnetometers and accelerometers, effectively upgrading the overall wellbore attitude accuracy of MWD systems [35]. Zhou et al. proposed a Backpropagation (BP) neural network-based algorithm for MWD installation error correction [36]. Bypassing the need for explicit installation error modeling or supplementary parameter calculations, this approach directly predicts attitude angles via ellipsoid fitting of raw measurement data, thereby effectively mitigating the impact of misalignment errors.

The application of AI algorithms to improve wellbore trajectory accuracy still relies heavily on model precision. Training these model parameters requires large dataset samples, placing high demands on hardware performance. Restricted by downhole tool dimensions, power consumption, and hardware architecture, AI-based trajectory improvement methods face certain limitations in real-time on-tool implementation. Furthermore, AI algorithms currently focus more on optimizing data integrity across the entire drilling cycle and removing data outliers; further technical breakthroughs are still needed regarding direct improvements to wellbore trajectory accuracy. Currently, the mainstream engineering methods for enhancing wellbore trajectory accuracy remain focused on magnetic interference correction, magnetometer and accelerometer error calibration, and sensor noise reduction. However, when drilling through high magnetic interference zones (such as vertical or cluster well sections), gyroscopic survey tools must be relied upon to complete the measurement.

### 2.3. Gyro-Based Wellbore Attitude Measurement Method

Gyroscopes are widely used in vehicles, ships, aircraft, missiles, and other carriers, serving as core sensors for attitude measurement, control, navigation, and positioning. Gyroscope types can be classified into mechanical gyros, optical gyros, and MEMS gyros, with optical gyros further divided into ring laser gyros and fiber optic gyros.

Mechanical gyroscopes incorporate internal high-speed rotors, making them highly susceptible to vibration and requiring absolute stationarity to achieve accurate azimuth measurements. Conversely, optical and MEMS gyroscopes—collectively termed solid-state gyroscopes—contain no moving parts, thereby broadening their operational versatility in wellbore trajectory measurement. Researchers have investigated wellbore trajectory measurement methods utilizing 9-axis sensor suites composed of MEMS inertial measurement units (IMUs) and magnetometers. In these integrated measurement architectures, magnetometers provide the initial attitude reference, while MEMS gyroscopes update quaternions or Euler angles in real time during dynamic operations to ensure continuous trajectory tracking. The algorithmic framework generally centers on a complementary filter scheme for integrated attitude determination [37,38].

Although ring laser gyroscopes (RLGs) offer high precision and serve as primary sensors for navigating large platforms (such as vessels and aircraft), their bulky physical footprint hinders integration into space-constrained drilling tools. As fiber optic gyroscope (FOG) fabrication techniques mature, medium-to-high-precision FOGs are increasingly miniaturized, enabling technological enhancements in both wireline gyro inclinometers and wireless gyro measurement-while-drilling (GMWD) systems. Extensive literature indicates that various FOG-based inclinometers and MWD systems have been successfully developed. For example, a research team at Beihang University developed FOG-based inclinometers with varied outer diameters that maintain absolute azimuth errors within 1°, fully satisfying conventional drilling requirements [39,40]. A standard FOG-MWD configuration integrates a triaxial FOG and a triaxial quartz flexural accelerometer to achieve full-attitude capability. However, its temperature tolerance remains a bottleneck, constraining its application primarily to shallow formations and vertical well sections [41,42].

The principal characteristics and downhole applicability of the different gyroscope types discussed above are summarized in Table 2.

Gyroscope-based wellbore attitude measurement methods and technologies are primarily developed within the framework of inertial navigation systems (INS). During continuous measurement, the angular velocity component output by the gyroscope—after removing the angular-rate component caused by carrier motion—tracks the attitude angles via quaternion updates. At static survey stations, the attitude matrix (Euler angle matrix) is derived using the dual-vector attitude determination principle to resolve attitude information. Within this mathematical framework, however, gyroscope and accelerometer errors inevitably couple into the attitude angles, causing error accumulation over time until divergence occurs. Under harsh drilling vibration environments, these sensor errors are further amplified, leading to severe measurement inaccuracies. Consequently, research efforts have largely focused on mitigating sensor error magnitudes in dynamic environments to improve attitude measurement precision.

Within the inertial-navigation framework, Zhang et al. developed a dynamic Allan variance-based model to characterize vibration-induced noise in fiber-optic gyroscopes (FOGs) used in MWD systems. The proposed method reduced the azimuth error by approximately 50% under random-vibration conditions [43]. To compensate for nonlinear inertial-sensor errors induced by periodic and random vibrations, Wang et al. developed a 36th-order extended Kalman filter (EKF) state model [44]. From a signal-processing perspective, Yang et al. developed a Synergy-enhanced Magpie Optimizer (SMO) to mitigate drilling-induced gyroscope errors. The algorithm enables online identification of MEMS gyroscope error parameters and reduces the associated errors; however, its improvement in azimuth accuracy was not quantitatively evaluated [45]. To mitigate the effects of high temperatures and mechanical shocks on MEMS gyroscopes, Yan et al. developed a signal-processing and north-finding method incorporating a drift-correction strategy, thereby improving the azimuth accuracy of downhole inclinometers [46]. Other studies have combined filtering algorithms with additional hardware to estimate and compensate for sensor errors. Ursenbach et al. proposed an in-drilling alignment algorithm that requires additional sensors to accurately measure axial displacement [47]. To reduce positioning errors, Li et al. developed an Augmented Fast Orthogonal Search/Kalman Filtering (FOS/KF) scheme to improve attitude and position estimation; however, the method requires an additional motorized rotational-modulation unit [48]. Based on the principle of rotational inertial navigation systems (RINSs), Sha et al. designed a MEMS-based MWD architecture incorporating a rotational-modulation unit and an axial speed encoder, with a Kalman filter used for azimuth estimation [49]. In addition, Yang et al. proposed an equivalent reverse-rotation modulation method combined with a robust iterative filtering scheme to identify gyroscope error parameters and improve wellbore trajectory measurement accuracy [50]. The principal research approaches to gyroscope-based wellbore attitude measurement are summarized in Table 3.

#### 2.3.1. Wellbore Attitude Measurement via Reduced Inertial Measurement Unit

For inclinometers or MWD systems configured with a single-axis gyroscope, the underlying measurement principle is categorized within inertial navigation as north-seeking. Such systems typically consist of a single-axis gyroscope and a triaxial accelerometer—a configuration commonly employed in vehicle positioning and orientation. During ground transport, vehicles experience relatively small variations in pitch and roll; hence, determining only the heading angle is sufficient. To minimize hardware costs, this simplified architecture—known as a Reduced Inertial Measurement Unit (RIMU)—is adopted. The single-axis gyroscope is deployed to sense the angular velocity of Earth’s rotation, which necessitates a high-precision sensor grade. Using the two horizontal angles (inclination and gravity toolface angle) derived from the triaxial accelerometer, the angular velocity measured by the single-axis gyroscope is projected onto the horizontal plane. Because a trigonometric relationship exists between the north component of Earth’s rotation rate and the projected horizontal angular velocity, the azimuth angle can be explicitly resolved.

Assuming the instrument is in a completely horizontal state with a single-axis gyroscope aligned along the longitudinal tool axis (*y*-axis), the angle between the tool axis and geographic north represents the azimuth angle (*az*). The relationship between the gyroscope output and the projected component of Earth’s rotation rate is formulated as follows:

*ω_y_* = *ω_ie_cosLat* ⋅ *cosaz* + *ε* = *ω_N_cosaz* + *ε* + *w*, (6)

where *ω_y_* denotes the actual output of the gyroscope, *ω_ie_* is Earth’s angular rotation rate, *Lat* is the local geographic latitude, *ω_N_* represents the north component of Earth’s rotation rate, *ε* is the residual gyro bias error, and *w* denotes the measurement random noise. Solving the equation above yields the explicit expression for the azimuth angle as follows:

(7) $$\mathrm{az}=\mathrm{atan}2(\sqrt{{\omega_{N}}^{2}-{\left(\omega_{y}-\varepsilon \right)}^{2}},\omega_{y}-\varepsilon ).$$

When a low-accuracy single-axis gyroscope is employed for azimuth measurement, its random noise and bias may substantially degrade the measurement accuracy, making it difficult to satisfy engineering requirements. To mitigate these effects, multi-position alignment is commonly performed at survey stations to improve the accuracy of wellbore attitude measurements. Moreover, when the instrument is in a non-horizontal orientation, measurement errors in the inclination and toolface angles are propagated into the azimuth solution. Based on the attitude matrix defined above, the output of the gyroscope mounted along the Y*_b_*-axis of the body frame can be expressed as follows:

*ω_y_* = *ω_N_* ⋅ *sininc* ⋅ *cosaz* − *ω_U_* ⋅ *cosinc* + *ε*, (8)

where *ω_U_* represents the vertical (upward) component of Earth’s angular rotation rate. Consequently, the principal-value estimate of the azimuth angle obtained from this single measurement can be expressed as follows:

(9) $$\mathrm{az}=\arccos\left(\frac{\omega_{y}+\omega_{U}\cdot \mathrm{cosinc}-\varepsilon }{\omega_{N}\cdot \mathrm{sininc}}\right),$$

It should be noted that a single-axis gyroscope has inherent limitations in azimuth determination. Mathematically, a measurement at a single position provides only one projection of the Earth’s angular rotation rate onto the gyroscope sensitive axis and therefore cannot uniquely determine the azimuth. Physically, different headings that are symmetric with respect to the gyroscope sensitive axis may produce identical outputs. To eliminate this ambiguity, multi-position indexing measurements are required. The magnitude, sign, and relationships of the gyroscope outputs at different indexed positions can then be jointly used to identify the azimuth quadrant and obtain a unique azimuth solution within the range [0°,360°).

**Figure 5:**
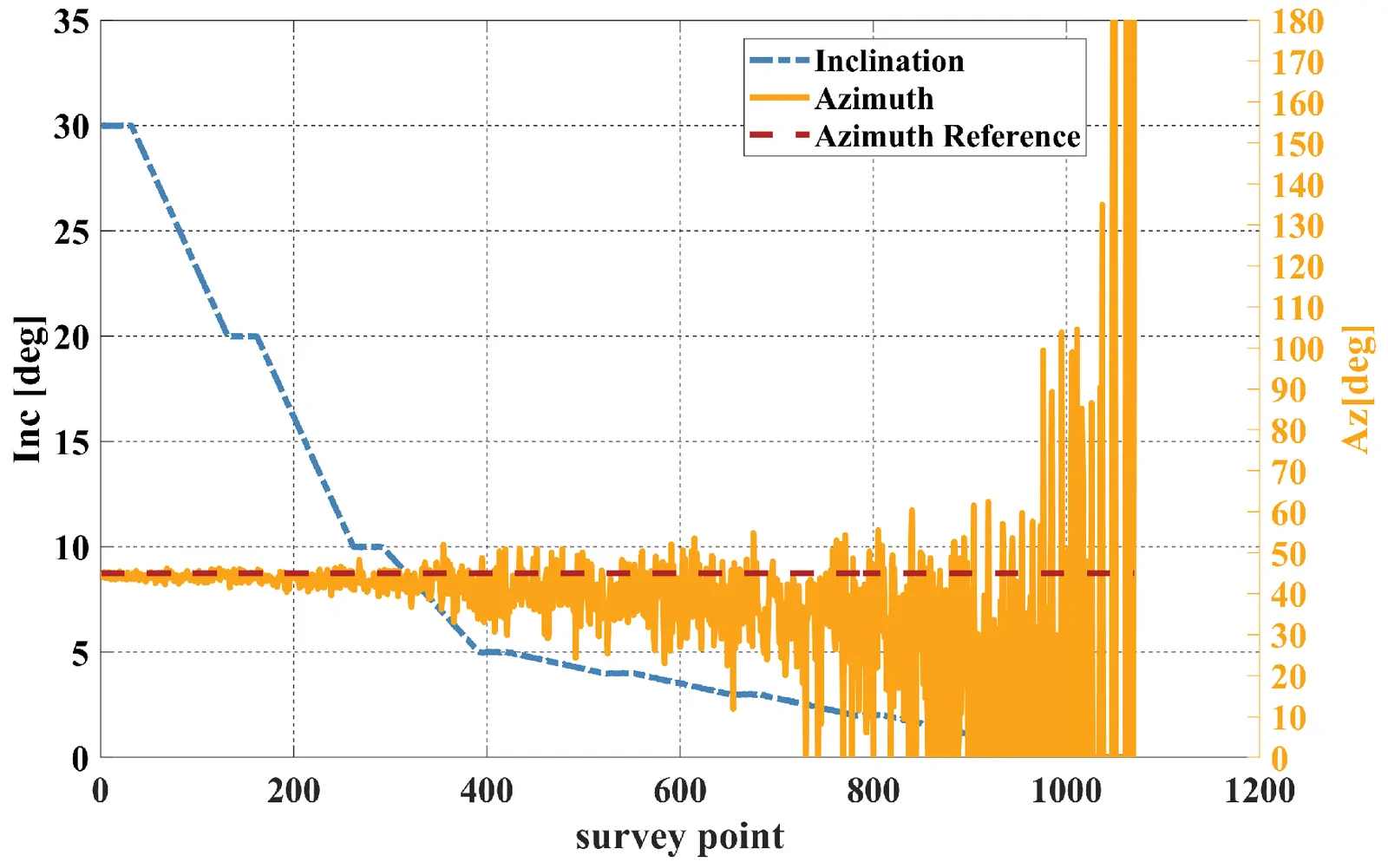
Azimuth measurement accuracy of the single-axis gyroscope-based measurement scheme over the inclination range of 0°~30°.

Azimuth accuracy is inherently constrained by inclination angle, toolface angle, and sensor errors; when the true angular velocity signal sensed by the gyroscope accounts for a low signal-to-noise ratio, calculation precision degrades significantly. Under conventional Euler angle definitions, the instrument orientation corresponds to an inclination angle *inc* = 0° in horizontal sections and *inc* = 90° in vertical sections. Consequently, resolving the azimuth under near-vertical conditions introduces a mathematical singularity into the azimuth formulation. Conversely, in horizontal well sections, the formulation simplifies directly to Equation (5), yielding the highest azimuth determination precision, albeit still governed by the residual gyro bias. Based on the sensor error parameters specified in Table 4, the simulated degradation trend of azimuth accuracy as the inclination decreases from 30° to 0° is illustrated in Figure 5.

The sensor accuracies listed in the table satisfy the operational precision requirements for wellbore attitude measurement. Because conventional inclination conditions do not induce azimuth accuracy degradation, Figure 5 depicts the calculated azimuth results only under selected low-inclination angles. The pronounced oscillation amplitude observed in the azimuth curves stems from the combined effects of random noise in both the accelerometers and the gyroscope. As the wellbore approaches a near-vertical state, the oscillation amplitude of the azimuth increases progressively. This degradation occurs because the horizontal projection component of Earth’s rotation sensed by the gyroscope becomes extremely weak under near-vertical conditions; when coupled with gyro random noise and residual bias error, it renders precise determination of the true heading impossible.

To mitigate the influence of gyroscope errors on azimuth determination, multi-position north-seeking methods are commonly employed in engineering applications. Owing to the confined downhole space, multi-position indexing at a survey station is generally restricted to rotation about the longitudinal drilling axis Y*_b_*. Therefore, the sensitive axis of the single-axis gyroscope should be aligned with either of the radial axes, X*_b_* or Z*_b_*, both of which are perpendicular to Y*_b_*. Taking a single-axis gyroscope mounted along the Z*_b_*-axis as an example, a four-position north-seeking method is described below. Let *ω*_1_ denote the gyroscope output at the initial position. The drilling tool is then rotated about its longitudinal Y*_b_*-axis in the same direction to angular positions of 90°, 180°, and 270° relative to the initial position. The corresponding gyroscope outputs are denoted by *ω*_2_, *ω*_3_, and *ω*_4_, respectively. The gyroscope outputs at the four indexing positions can be expressed as follows:

(10) $$\{\begin{array}{l} \omega_{1}={-\omega }_{N}\cdot \sin\mathrm{az}\cdot \sin\mathrm{tf}+\omega_{N}\cdot \cos\mathrm{az}\cdot \cos\mathrm{inc}\cdot \cos\mathrm{tf}+\omega_{U}\cdot \sin\mathrm{inc}\cdot \cos\mathrm{tf}+\varepsilon +\varepsilon_{r1}\\ \omega_{2}={-\omega }_{N}\cdot \sin\mathrm{az}\cdot \cos\mathrm{tf}-\omega_{N}\cdot \cos\mathrm{az}\cdot \cos\mathrm{inc}\cdot \sin\mathrm{tf}-\omega_{U}\cdot \sin\mathrm{inc}\cdot \sin\mathrm{tf}+\varepsilon +\varepsilon_{r2}\\ \begin{array}{l} \omega_{3}=\omega_{N}\cdot \sin\mathrm{az}\cdot \sin\mathrm{tf}-\omega_{N}\cdot \cos\mathrm{az}\cdot \cos\mathrm{inc}\cdot \cos\mathrm{tf}-\omega_{U}\cdot \sin\mathrm{inc}\cdot \cos\mathrm{tf}+\varepsilon +\varepsilon_{r3}\\ \omega_{4}=\omega_{N}\cdot \sin\mathrm{az}\cdot \cos\mathrm{tf}+\omega_{N}\cdot \cos\mathrm{az}\cdot \cos\mathrm{inc}\cdot \sin\mathrm{tf}+\omega_{U}\cdot \sin\mathrm{inc}\cdot \sin\mathrm{tf}+\varepsilon +\varepsilon_{r4} \end{array} \end{array}.$$

When the positions are separated by 180°, subtracting the gyroscope outputs between the two opposing positions effectively cancels out the constant gyro bias error, yielding *ε_r_*_1_ ≈ *ε_r_*_3_ and *ε_r_*_2_ ≈ *ε_r_*_4_ Furthermore, these differential expressions can be reformulated as follows:

(11) $$\frac{\omega_{4}-\omega_{2}}{\omega_{3}-\omega_{1}}=\frac{\omega_{N}\cdot (\sin\mathrm{az}\cdot \cos\mathrm{tf}+\cos\mathrm{az}\cdot \cos\mathrm{inc}\cdot \sin\mathrm{tf})+\omega_{U}\cdot \sin\mathrm{inc}\cdot \sin\mathrm{tf}}{\omega_{N}\cdot (\sin\mathrm{az}\cdot \sin\mathrm{tf}-\cos\mathrm{az}\cdot \cos\mathrm{inc}\cdot \cos\mathrm{tf})-\omega_{U}\cdot \sin\mathrm{inc}\cdot \cos\mathrm{tf}}.$$

Combining the differential expressions above, the trigonometric components of the azimuth angle can be derived, yielding the explicit azimuth expression as follows:

(12) $$\mathrm{az}=\mathrm{atan}2\left[\frac{(\omega_{3}-\omega_{1})\mathrm{sintf}+(\omega_{4}-\omega_{2})\mathrm{costf}}{2\omega_{N}},\frac{(\omega_{1}-\omega_{3})\mathrm{costf}+(\omega_{4}-\omega_{2})\mathrm{sintf}-2\omega_{U}\mathrm{sininc}}{2\omega_{N}\mathrm{cosinc}}\right].$$

Dual-axis gyroscope configurations can likewise utilize the multi-position indexing method of single-axis gyroscopes to resolve the azimuth angle; however, they remain incapable of executing continuous dynamic attitude determination. An alternative azimuth determination method is presented below. Typically, dual-axis gyroscopes are mounted along the radial axes of the downhole instrument (specifically, the *x*-axis and *z*-axis), leaving the longitudinal tool axis without gyroscope coverage. Incorporating the attitude transformation matrix, the mathematical expressions are formulated as follows:

(13) $$\{\begin{array}{l} \omega_{x}=\omega_{N}\left(-\sin\mathrm{az}\cos\mathrm{tf}-\cos\mathrm{azcosinc}\sin\mathrm{tf}\right)-\omega_{U}\sin\mathrm{inc}\sin\mathrm{tf}\\ \omega_{z}=\omega_{N}\left(-\sin\mathrm{az}\sin\mathrm{tf}+\cos\mathrm{azcosinc}\cos\mathrm{tf}\right)+\omega_{U}\sin\mathrm{inc}\cos\mathrm{tf} \end{array}$$

Furthermore, the explicit analytical expression for the azimuth angle can be derived as follows:

(14) $$\mathrm{az}=\mathrm{atan}2\left(-\frac{{\omega_{x}\cos\mathrm{tf}+\omega }_{z}\sin\mathrm{tf}}{\omega_{N}},\frac{{\omega_{z}\cos\mathrm{tf}-\omega }_{x}\sin\mathrm{tf}-\omega_{U}\sin\mathrm{inc}}{\omega_{N}\cos\mathrm{inc}}\right)$$

**Figure 6:**
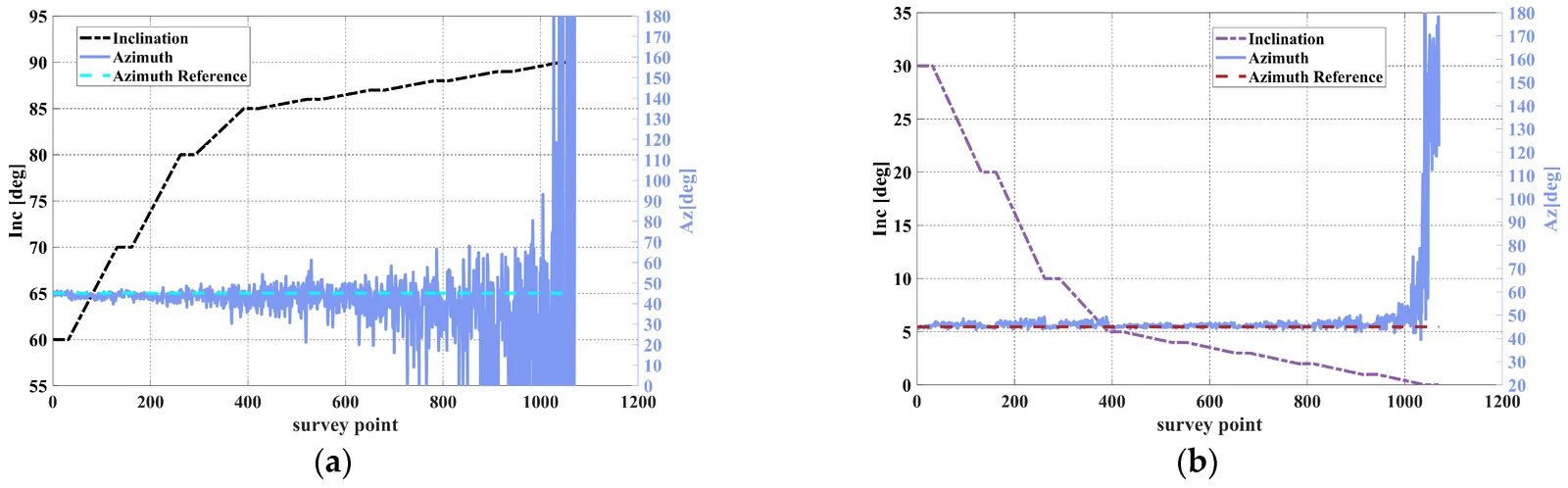
Azimuth accuracy analysis of a dual-axis gyroscope in the 90°~0° inclination range. (**a**) Azimuth error amplitude characteristics as the inclination angle approaches 90°. (**b**) Azimuth error amplitude characteristics as the inclination angle approaches 0°.

As indicated by the denominator in the equation, when performing azimuth measurements in horizontal well sections (where inclination *inc* = 90° under Euler angle sequence rotations), the azimuth calculation approaches a mathematical singularity or becomes unmeasurable. This structural singularity explains why certain gyroscopic tool configurations fail to operate in horizontal wells. Furthermore, dual-axis gyroscopes—similar to triaxial gyroscopes—still face measurement dead zones in near-vertical well sections. Based on the sensor error parameters specified in Table III, the simulated variation trends of the azimuth angle derived from a dual-axis gyroscope under near-horizontal and near-vertical conditions are depicted in Figure 6a and Figure 6b, respectively. Under near-vertical conditions (*inc* = 0°, cos*inc =* 1, sin*inc* = 0), Equation (13) shows no mathematical singularity; nevertheless, constrained by the determination accuracy of the toolface angle, the azimuth angle in near-vertical sections remains unresolvable with high precision—a limitation that will be discussed further in the subsequent section.

#### 2.3.2. Wellbore Attitude Measurement via Complete Inertial Measurement Unit

Under a triaxial gyroscope and triaxial accelerometer configuration, the system provides three-degree-of-freedom angular velocity and linear acceleration measurements, which is referred to as a Complete Inertial Measurement Unit (CIMU) in inertial navigation. This configuration enables full-attitude trajectory determination for downhole drilling tools. The mathematical relationship between the actual output of each gyroscope axis and the corresponding components of Earth’s rotation rate is expressed as follows:

(15) $$\{\begin{array}{c} \omega_{x}=\omega_{N}\left(\sin\mathrm{az}\cos\mathrm{tf}+\cos\mathrm{az}\cos\mathrm{inc}\sin\mathrm{tf}\right)-\omega_{U}\sin\mathrm{inc}\sin\mathrm{tf}\\ \omega_{y}=\omega_{N}\cos\mathrm{az}\sin\mathrm{inc}+\omega_{U}\cos\mathrm{inc}\\ \omega_{z}=\omega_{N}\left(\sin\mathrm{az}\sin\mathrm{tf}-\cos\mathrm{az}\cos\mathrm{inc}\cos\mathrm{tf}\right)+\omega_{U}\sin\mathrm{inc}\cos\mathrm{tf} \end{array}$$

By algebraic transformation of the equation above, the explicit expression for the azimuth angle under the triaxial gyroscope measurement scheme is derived as follows:

*az* = atan2(*ω_x_*cos*tf* + *ω_z_*sin*tf*,(*ω_x_*sin*tf* − *ω_z_*cos*tf*)cos*inc* + *ω_y_*sin*inc*) (16)

Theoretically, provided that the gyroscopes and accelerometers possess sufficient accuracy, a CIMU equipped with triaxial gyroscopes and triaxial accelerometers can achieve full-inclination attitude determination across all wellbore sections. However, when the tool approaches a vertical orientation, the signal-to-noise ratio (SNR) of the transverse accelerometers deteriorates significantly, degrading the determination precision of the gravity toolface angle, which subsequently degrades the azimuth accuracy. Expressing the toolface angle in an error-containing form yields:

(17) $$\mathrm{tf}=\arctan\left(\frac{-g_{x}+\delta_{x}}{g_{z}+\delta_{z}}\right)$$

**Figure 7:**
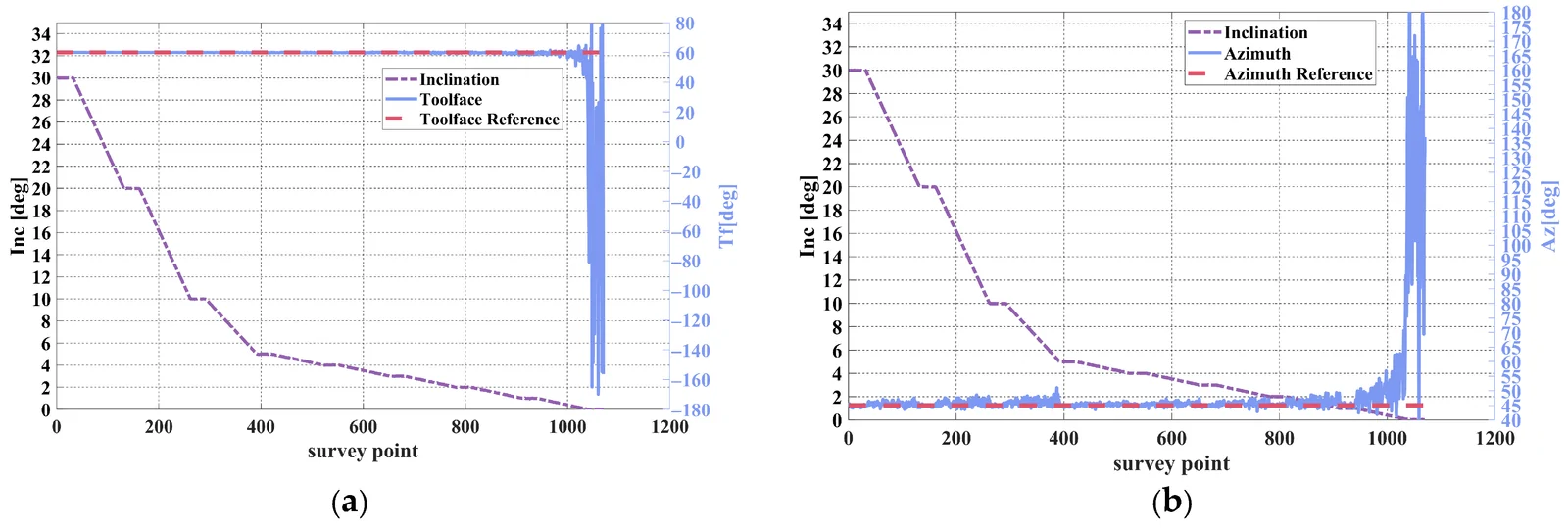
Impact of accelerometer bias on attitude accuracy in the 30°~0° inclination range. (**a**) Error variation curve of gravity toolface angle as inclination approaches 0°. (**b**) Error variation curve of azimuth angle as inclination approaches 0°.

Under near-vertical conditions, the radial accelerometers sense minimal gravity acceleration components. In extreme scenarios where both radial accelerometers yield output values near zero, substantial uncertainty is introduced into the determination of the gravity toolface angle. When the instrument is fully vertical, the radial accelerometer outputs consist purely of noise, triggering random phase jumps in the calculated toolface angle. Based on the sensor error parameters specified in Table 4, the variations in toolface angle and azimuth angle as the inclination angle decreases from 30° to 0° were simulated. As illustrated in Figure 7a, the toolface angle error increases progressively with decreasing inclination until divergence occurs. Coupled with the analysis from Equation (15), the accuracies of the toolface angle and inclination angle directly govern the determination precision of the azimuth angle; consequently, as the inclination angle decreases, the oscillation amplitude of the measured azimuth angle grows progressively larger (as depicted in Figure 7b).

**Figure 8:**
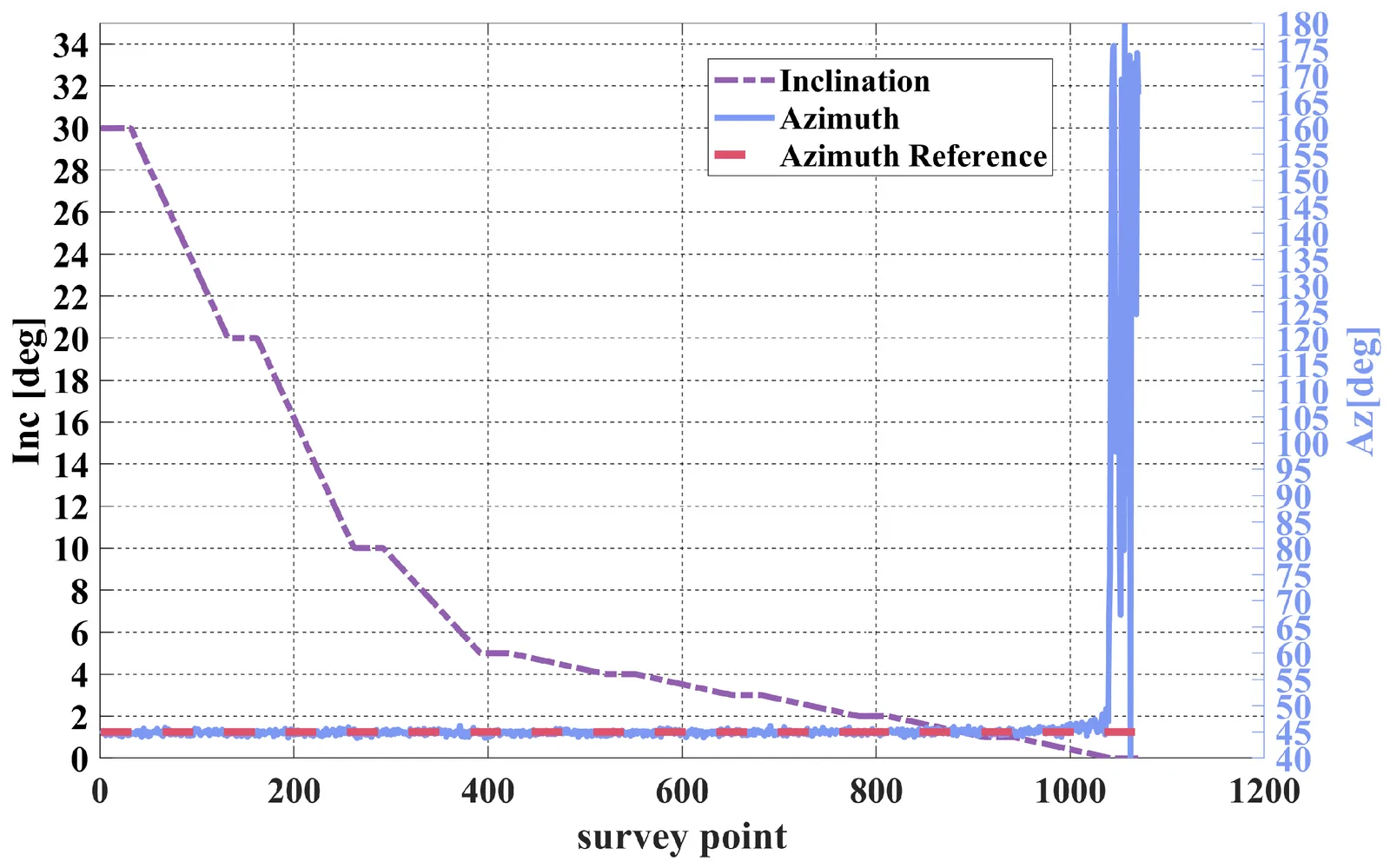
Azimuth accuracy in the 30°~0° inclination range under low accelerometer error conditions.

If the accelerometer accuracy is sufficiently high, the error divergence of the azimuth angle in near-vertical well sections will be mitigated; however, the mathematical singularity in purely vertical sections remains unavoidable. As shown in Figure 8, with the gyroscope precision kept constant and the bias and random noise of the accelerometer reduced to 10 μg, the azimuth angle exhibits singularity only near an inclination angle of 0°. Consequently, in near-vertical well sections, the gyro toolface angle is commonly used instead of the azimuth angle and gravity toolface angle to determine the downhole orientation of the tool string.

## 3. Wellbore Trajectory Measurement Technology

**Figure 9:**
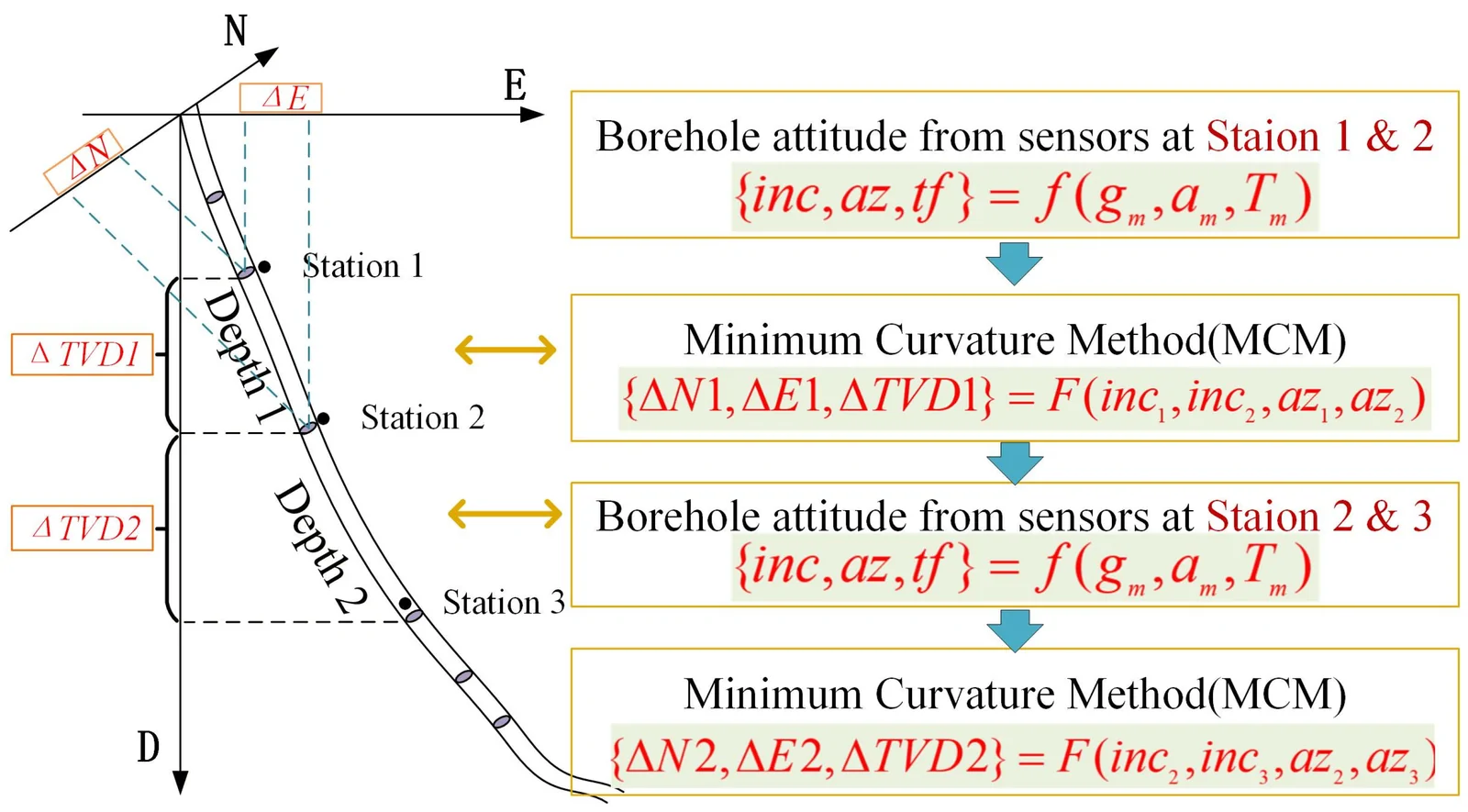
Schematic diagram of the wellbore trajectory reconstruction method.

Ground or airborne vehicles can rely on multi-source information—such as inertial navigation systems, laser velocimeters, and Global Navigation Satellite Systems (GNSS)—for real-time trajectory measurement and tracking. In drilling operations, however, due to the scarcity of information and harsh downhole environments, the real-time trajectory of the instrument cannot be directly constructed. Instead, the wellbore trajectory must be back-calculated using the downhole attitudes measured by sensors alongside the drill pipe or cable length. Specifically, as the instrument reaches each survey station, sensors measure the azimuth and inclination angles at that station, while the distance between two adjacent stations is obtained by subtracting their respective measured depths (MD). It is then assumed that the segment between two survey stations follows a curve or straight line that can be mathematically characterized. The azimuth and inclination angles of the two stations reflect the spatial orientation of this curve. By projecting the spatial curve onto a reference coordinate frame, the easting, northing, and vertical components are derived. The Minimum Curvature Method (MCM) is the most widely adopted approach for wellbore trajectory reconstruction; owing to its simple computation and reasonable assumptions, it serves as the current industry standard. Other methods include the radius of curvature method, average angle method, and balanced tangential method, all of which exhibit certain limitations [51,52,53]. A schematic diagram of wellbore trajectory calculation and reconstruction is shown in Figure 9.

### 3.1. Discrete Wellbore Trajectory Reconstruction Method

**Figure 10:**
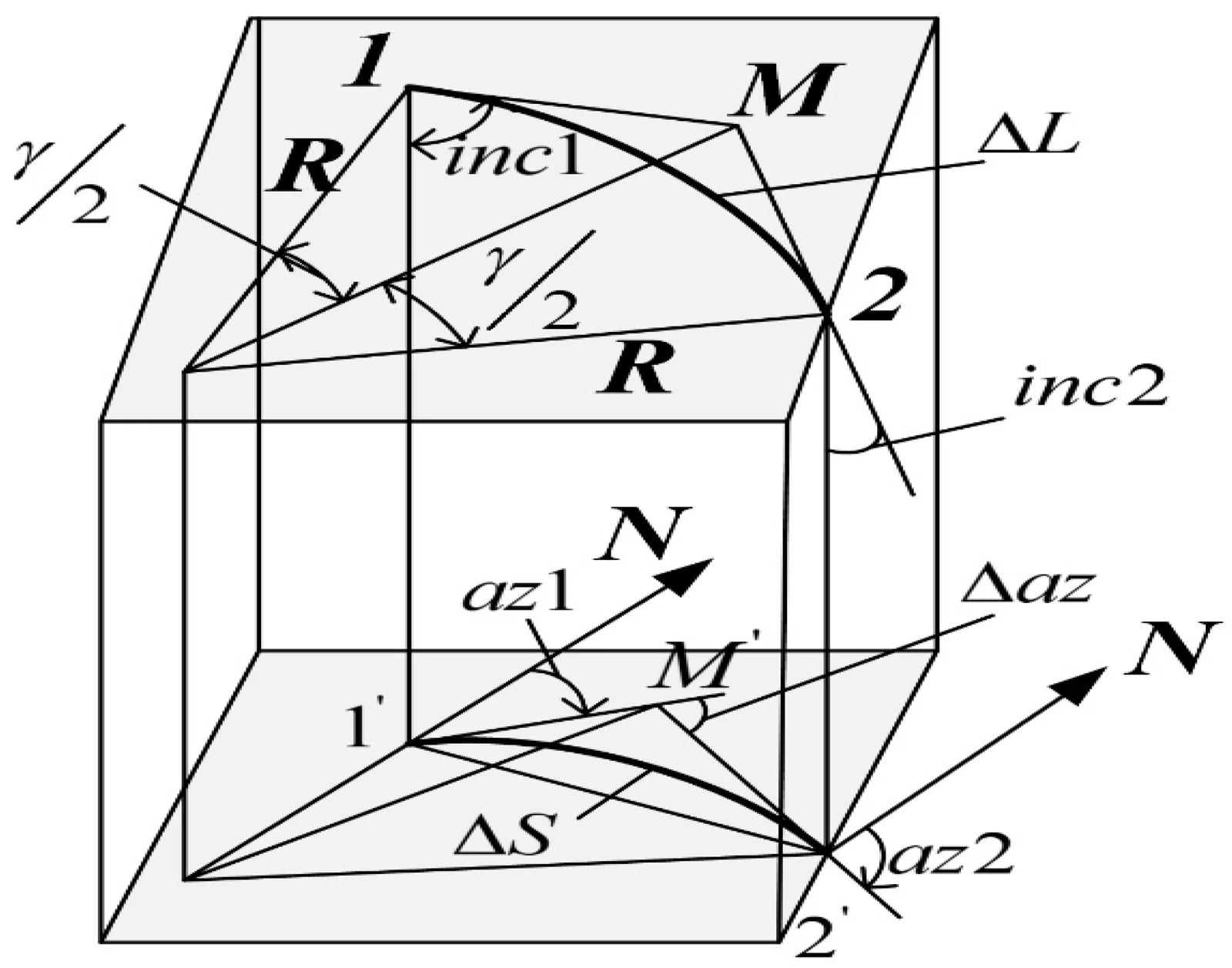
Schematic Diagram of the Spatial Geometry Assumption of the MCM.

The mainstream scheme adopted in current wellbore trajectory measurement is the discrete survey station-based trajectory reconstruction method, with the Minimum Curvature Method proposed by Wilson serving as the primary computational model [54]. Subsequently, relevant calculation models were refined on the basis of the MCM to render the underlying assumptions more comprehensive [55]. Additionally, Prof. Jiying Zheng from China proposed a wellbore trajectory calculation model that assumes the section between two survey stations to be a cylindrical helix; due to its conceptual accuracy and clear formulation, it is also a widely used trajectory calculation method [56]. Furthermore, Eren et al. detailed an “Improved Tangential Method” in the literature for directional wellbore trajectory calculation, which is characterized by its applicability to both straight intervals and trajectories with arbitrary tortuosity [57]. The accuracy of discrete trajectory reconstruction methods and computational models is significantly sensitive to the station spacing, which in engineering practice is typically defined by the length of a single drill pipe stand (approximately 27 m). As shown in Figure 10, this phenomenon is analyzed by taking the Minimum Curvature Method as a representative model.

The computational model of the MCM is formulated as follows:

(18) $$\{\begin{array}{c} \begin{array}{c} \begin{array}{c} \gamma =\arccos\left(\cos{\mathrm{inc}}_{1}\cos{\mathrm{inc}}_{2}+\sin{\mathrm{inc}}_{1}\sin{\mathrm{inc}}_{2}\cos\Delta \mathrm{az}\right)\\ \lambda_{M}=\left(180\Delta L\tan(\gamma /2)\right)/\left(\pi \gamma \right) \end{array}\\ \Delta Z=\lambda_{M}\left(\cos{\mathrm{inc}}_{1}+\cos{\mathrm{inc}}_{2}\right) \end{array}\\ \Delta X=\lambda_{M}\left(\sin{\mathrm{inc}}_{1}\cos{\mathrm{az}}_{1}+\sin{\mathrm{inc}}_{2}\cos{\mathrm{az}}_{2}\right)\\ \begin{array}{c} \Delta Y=\lambda_{M}\left(\sin{\mathrm{inc}}_{1}\sin{\mathrm{az}}_{1}+\sin{\mathrm{inc}}_{2}\sin{\mathrm{az}}_{2}\right)\\ \Delta S\approx \lambda_{M}\left(\sin{\mathrm{inc}}_{1}+\sin{\mathrm{inc}}_{2}\right)\frac{\pi \Delta \mathrm{az}}{360\tan(\Delta \mathrm{az}/2)} \end{array} \end{array},$$

where *γ* is the dogleg angle; *λ_M_* is an intermediate variable; Δ*Z*, Δ*X*, Δ*Y*, and Δ*S* represent the vertical, northing, easting, and horizontal displacement increments, respectively; Δ*L* is the measured depth increment between the two survey stations; *inc*_1_ and *inc*_2_ are the inclination angles at the two survey stations; and *az*_1_ and *az*_2_ are the corresponding azimuth angles. As indicated by these equations, the wellbore attitude angles at the two survey stations determine the spatial curvature (dogleg angle *γ*) between them. Because the radius of curvature is assumed to be constant, detailed local trajectory variations between adjacent survey stations cannot be resolved. This survey station spacing introduces significant calculation errors in curved well segments. Indeed, Codling et al. highlighted in the literature that “a smaller station spacing yields calculation results closer to the true trajectory.” [58] Therefore, reducing the survey-station spacing or adding more actual measurement stations during drilling to increase attitude-sampling density is generally considered an effective way to improve trajectory accuracy. However, the spacing between survey stations is usually determined by the drill-pipe length, and virtual survey stations can also be generated using interpolation. The attitude angles at these interpolated stations, however, are estimated solely from the original measurements; they can only make the estimated trajectory smoother and do not provide new observational information. To mitigate the undersampling associated with conventional methods that acquire measurements only during pipe connections, ElGizawy et al. proposed the Definitive Dynamic MWD Surveys (DDS) method, which enables high-density wellbore attitude measurements during drilling [59]. To ensure measurement accuracy, an error-compensation model adapted to dynamic drilling conditions was developed.

During drilling operations across formations with diverse lithological characteristics, harsh downhole working conditions cause the actual drilled trajectory to deviate from ideal geometric patterns, despite strict shape constraints imposed during well path design. To better reflect the true downhole path, researchers have developed trajectory prediction and optimization methods. The core strategy incorporates drilling uncertainties into optimization models, leveraging geometrical frameworks to render the calculated trajectories closer to reality. For instance, considering parameter uncertainties, Huang et al. proposed a multi-objective trajectory optimization method for the drilling process, establishing a probability density function to characterize wellbore tortuosity based on actual field data analysis [60]. Wang et al. introduced a Snake Optimizer (SO) algorithm for trajectory computation, incorporating trajectory length, drag, and torque into a multi-factor weighting function for optimization [61]. Gao et al. developed an L2-SSA-LSTM neural network architecture to predict the wellbore trajectory at the next time step by analyzing sensor data, attitude information, drag, and other survey parameters; their model fuses these predictions with analytical calculations in real time to yield optimal trajectory parameters [62]. Furthermore, Huang et al. proposed a novel trajectory prediction model based on an LSTM framework, which constructs the wellbore trajectory using inclination and azimuth data without relying on spatial shape or geometric assumptions [63].

Whether employing wellbore trajectory calculation models based on traditional geometric assumptions or prediction models built upon AI algorithmic frameworks, both approaches exhibit a high degree of dependence on downhole sensor data and attitude angles recorded at individual survey stations. For geometric assumption methods, complex drilling environments introduce substantial uncertainty regarding the actual path traversed by the drilling assembly. Consequently, increasing the frequency and spatial density of measurements is critical to enhancing the accuracy of wellbore trajectory reconstruction. Conversely, AI-driven trajectory prediction models rely heavily on data-driven paradigms to establish relatively accurate mathematical formulations, presenting a notable disadvantage in terms of computational overhead. Nevertheless, such methods extend beyond conventional wellbore attitudes by integrating multi-source information, including geological characteristics, drill string mechanics, and drill bit-rock interaction mechanics. This multidimensional integration enables the optimization of trajectory curves that conform more closely to actual drilling paths under conditions of trajectory uncertainty. The synergistic combination of physical modeling and data-driven learning endows these AI frameworks with superior application prospects, aligning seamlessly with the future developmental trajectory of intelligent and automated drilling systems.

### 3.2. Continuous Wellbore Trajectory Measurement Method

Two concepts should be distinguished in continuous wellbore trajectory measurement. The first is trajectory reconstruction based on continuously measured attitude angles and measured depth (MD). In this approach, inclination and azimuth determine the direction of the wellbore, whereas the displacement increment is obtained from independently measured depth or rate-of-penetration information. Although increasing the attitude-sampling density can improve the temporal and spatial resolution of the reconstructed trajectory, this approach does not constitute fully autonomous positioning because it still relies on external depth measurements. The second concept is autonomous inertial positioning, in which attitude, velocity, and position are estimated directly by integrating the outputs of gyroscopes and accelerometers. This section focuses primarily on the latter and reviews its error-accumulation mechanism and engineering limitations.

In other fields, inertial navigation, terrain or geomagnetic matching navigation, visual navigation, and celestial navigation are commonly used for real-time trajectory estimation [64,65,66]. Because of the enclosed downhole environment and stringent hardware constraints, most external-reference navigation methods cannot currently be directly deployed in drilling tools. Inertial navigation systems (INSs), owing to their self-contained operation, therefore represent the most feasible core framework for autonomous downhole positioning. However, gyroscope and accelerometer errors propagate through successive integration: gyroscope errors affect attitude estimation, whereas accelerometer errors are integrated into velocity and subsequently into position. Consequently, even small sensor errors can cause the estimated trajectory to drift over time.

**Figure 11:**
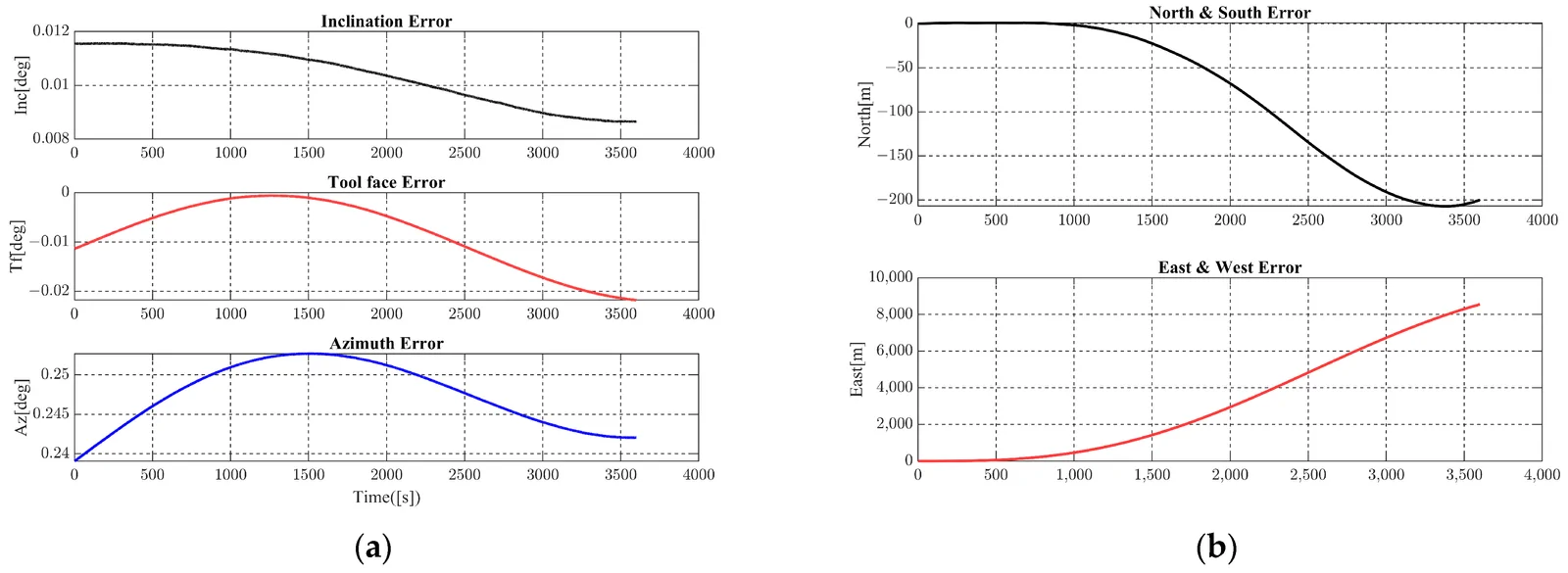
SINS trajectory component cumulative error over 1 h. (**a**) Attitude error curves over 1 h of static navigation; (**b**) eastward and northward displacement error curves over 1 h of static navigation.

Considering the requirements imposed by the drilling environment on inertial sensors and the navigation computation system, the conditions for the static navigation simulation are presented in Table 5. Without altitude damping, the vertical channel of an inertial navigation system diverges rapidly; therefore, it is typically combined with a barometer or altimeter to correct vertical errors. As a result, the vertical channel error curves are not presented in the simulation. As shown in Figure 11, regarding the wellbore attitude angles, the errors in azimuth (*az*) and horizontal attitude angles (*inc* and *tf*) remain small during the 1-h continuous solution process, satisfying downhole accuracy requirements. However, under 1-h static conditions, the trajectory errors in the east–west and north–south directions diverge severely.

During actual rotary drilling, if the drilling assembly rotates regularly and its rotational speed can be accurately obtained, rotational modulation inertial navigation algorithms can be employed to periodically modulate certain inertial sensor errors and improve the observability of the error states, thereby suppressing the accumulation of positioning and orientation errors to some extent. However, actual drilling operations are affected by axial, radial, and torsional vibrations, as well as stick–slip motion. Consequently, the rotational speed of the drilling assembly generally contains substantial fluctuations and high-amplitude noise, making it difficult to provide accurate and reliable motion constraints for the inertial navigation system. Although dynamic motion can improve the observability of some inertial errors, complex drilling disturbances also introduce additional error sources into the navigation computation process, potentially offsetting these observability gains and aggravating trajectory errors. Therefore, under complex drilling conditions, inertial navigation alone cannot meet the accuracy requirements for wellbore trajectory measurement.

Considering the drilling environment, Noureldin et al. analyzed the error propagation laws of the inertial navigation solution framework in greater detail in their study; however, much like integrated navigation concepts applied in other scenarios, providing the inertial navigation system with accurate drilling speed or displacement constraints during drilling can significantly suppress navigation errors [67]. For instance, when a drilling tool reaches a static survey station, a zero-velocity update (ZUPT) algorithmic framework can be adopted, which partially suppresses the bias errors of gyroscopes and accelerometers; nevertheless, it cannot effectively estimate and compensate for the equivalent drift gyro errors that directly affect azimuth accuracy. Jurkov et al. simulated the three-dimensional motion process of drilling in their paper; however, because drilling motion predominantly exhibits rotation around the longitudinal axis, the excitation effect of the motion on system errors is relatively weak, rendering the azimuth angle error incompletely observable [68]. Wang et al. utilized a reduced inertial measurement unit with a single-axis fiber optic gyroscope configuration. By leveraging drill pipe length and rate of penetration (ROP) information, they designed a wellbore trajectory survey filtering architecture and validated navigation attitude and position errors on a three-axis turntable; however, numerous limitations remain regarding its practical engineering application [69]. To enhance sensor observability, Ji et al. proposed a backtracking zero-velocity update method that constructs reverse navigation parameters by reading data stored in memory, indirectly increasing the time and rotation angle of the 3D motion to improve both observability and attitude accuracy; however, complete sensor observability remains unachieved, and this approach can only offer a methodical reference toward realizing continuous trajectory measurement [70].

**Figure 12:**
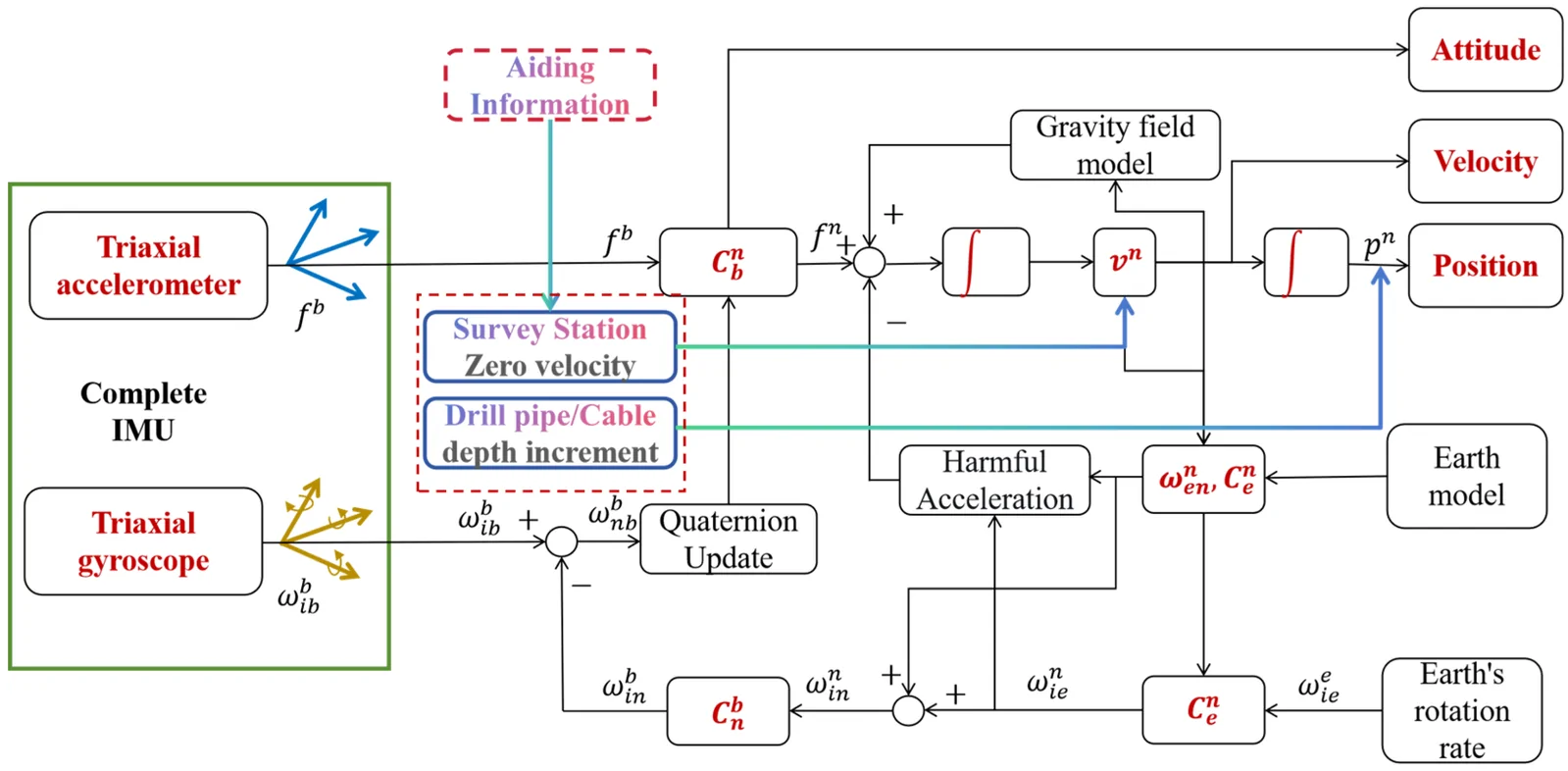
Trajectory measurement method based on the principles of inertial navigation.

In summary, a general framework for continuous wellbore trajectory measurement based on aided inertial navigation is illustrated in Figure 12. Zero-velocity constraints at survey stations and measured-depth increment constraints provided by drill pipes or cables constitute important auxiliary information for limiting inertial-navigation error accumulation. The zero-velocity information can be used to correct velocity errors and estimate certain inertial-sensor errors, whereas measured-depth increments, when combined with the tool attitude, can provide displacement constraints along the wellbore. However, these auxiliary observations still depend on existing engineering conditions, such as survey stations, drill pipes, or cables, and their effectiveness is limited by downhole vibration, stick–slip motion, and incomplete observability of the error states. Therefore, the transition from discrete survey-station-based trajectory reconstruction to continuous wellbore trajectory measurement continues to present substantial theoretical and engineering challenges.

## 4. Discussion

Underground energy exploration is shifting toward more complex environments such as deep-earth and deep-sea regions. Under current technological paradigms, to achieve more refined “underground navigation,” wellbore attitude and trajectory measurement technologies demonstrate a trend of diversified development: sensor measurement techniques are gradually shifting toward multi-sensor data fusion combining magnetometers, accelerometers, and gyroscopes to acquire detailed parameters of the wellbore or drilling tool in blind zones in greater detail; wellbore attitude measurement relies on abundant heterogeneous sensors, selectively adopting optimal measurement methods based on operational workflows to monitor drilling tool attitude; and wellbore trajectory measurement employs multiple technical pathways—including trajectory prediction, trajectory reconstruction, and trajectory optimization—to feed back more accurate wellbore curves to drilling tool control systems and surface platforms. Overall, wellbore attitude and trajectory measurement technologies are undergoing a transition from “static discrete measurement” to “dynamic continuous measurement.” However, achieving high-precision, highly reliable, all-scenario autonomous measurement and intelligent control still faces numerous theoretical and technical bottlenecks. Based on current research progress, future research priorities may revolve around the following directions:

1. Extreme thermodynamic environments during drilling impose higher demands on sensor technology upgrades and iterations. Magnetometers and mechanical gyroscopes offer the advantage of high-temperature resistance, but mechanical gyroscopes still suffer from reliability disadvantages under severe vibration environments. Fiber optic gyroscopes (FOGs) possess advantages such as high precision and vibration resistance, but cannot be applied in high-temperature scenarios. The physical models coupling downhole tool motion patterns with sensors still require further optimization. The laws governing how nonlinear errors impact sensors are difficult to accurately characterize through mathematical or physical models alone, whereas artificial intelligence algorithms based on data-driven approaches combined with physical models may remedy this shortcoming. Additionally, developing novel high-precision sensors—such as quantum vector magnetometers and quantum gravity gradiometers—to perceive subsurface physical field variations could provide effective matching points for subsurface navigation, forming a matching navigation technology framework similar to surface navigation.
2. Real-time continuous trajectory measurement technology in dynamic drilling environments is one of the effective ways to advance intelligent drilling and logging. To achieve true continuous trajectory measurement, inertial navigation represents the only self-contained technological scheme that can be assembled on drilling tools, yet its theoretical and methodological barriers have not been fundamentally resolved. Future research can seek breakthroughs in the following aspects: first, high-frequency, high-precision auxiliary information constraints, such as conducting research on integrated navigation and positioning methods leveraging kinematic constraints like drill pipes, rate of penetration (ROP), and cables to suppress the accumulation of trajectory calculation errors; second, inertial-based multi-source information constraint matching navigation, utilizing quantum sensing technology to establish gravitational and geomagnetic databases to form a continuous wellbore trajectory measurement methodology incorporating inertial measurement, quantum magnetic measurement, and quantum gravity gradient measurement; and third, wellbore trajectory measurement technology under artificial intelligence frameworks, integrating sensor data, physical models, drillstring dynamics, and other information into intelligent trajectory calculation and prediction frameworks to provide constraint conditions that better match real-world conditions for trajectory calculation.
3. Intelligent drilling measurement and closed-loop control jointly determine the autonomy level of the drilling tool in reaching the reservoir. Wellbore attitude and trajectory measurements provide critical input parameters for trajectory control. However, the target hitting rate in oil and gas reservoirs is also influenced by multiple factors such as downhole data transmission speed, geological models, and mechanical properties of the drilling tool. In the future, efforts should be directed toward the integrated research of underground navigation, control, and decision-making technologies. In addition, digital twin technology for wellbore trajectory measurement and control will facilitate virtual–reality interactive mapping of the drilling process, enabling intuitive feedback to the surface regarding the characteristic behaviors of the downhole drilling tool. Building an intelligent decision-making platform that integrates data-driven and physical models within the digital twin framework is expected to drive the transformation of drilling operations from “post-drilling analysis” to “real-time guidance and control during drilling.”

With the increasing demand for the exploration and development of deep-earth, deep-sea, unconventional oil and gas, and geothermal resources, wellbore attitude and trajectory measurement technologies are progressively evolving toward a higher-dimensional paradigm of “underground spatial positioning and orientation.” The ultimate vision is to establish an “underground positioning system” that parallels the ground-surface environment in terms of abundant informational constraints, thereby realizing continuous, high-precision positioning capabilities tailored to enclosed subsurface spaces.

## 5. Conclusions

This paper systematically examines the current state of wellbore attitude and trajectory measurement technologies in oil and gas drilling. It discusses their measurement principles and practical engineering workflows, with a comprehensive analysis of sensor configurations, attitude determination methods, and trajectory measurement approaches.

The review first examines wellbore attitude measurement based on magnetometer and gyroscope configurations. It analyzes the advantages and limitations of conventional magnetometer–accelerometer systems and, using mathematical models, explains how magnetic interference from the wellbore and drilling tools affects attitude accuracy. The analysis highlights that current research primarily focuses on improving magnetic azimuth accuracy through magnetometer error compensation. Mainstream methods for gyroscope–accelerometer-based inertial measurement are then reviewed, with current efforts focusing on sensor noise reduction, dynamic error suppression, and the mitigation of navigation errors through motion constraints. A comparison of the mathematical attitude models shows that RIMUs equipped with single- or dual-axis gyroscopes cannot achieve full-attitude determination across all inclination conditions. By contrast, a CIMU configuration incorporating a three-axis gyroscope can theoretically provide full-attitude measurement. However, in near-vertical well sections, the reduced signal-to-noise ratio of radial accelerometers degrades gravity toolface accuracy and, consequently, azimuth accuracy. This challenge affects both magnetometer- and gyroscope-based measurement schemes.

The review further examines wellbore trajectory measurement from two perspectives: discrete trajectory reconstruction and continuous trajectory measurement. The accuracy of discrete trajectory reconstruction is primarily limited by the spacing between survey stations. Increasing the number of actual survey stations and reducing the spacing between them provide denser attitude measurements, thereby improving reconstruction accuracy. In addition, by incorporating multi-source data acquired during drilling, artificial intelligence methods can assist in predicting and optimizing the trajectory between survey stations, producing a smoother curve that more closely approximates the actual wellbore trajectory. Continuous wellbore trajectory measurement remains a major technical bottleneck, and current research is conducted largely within the theoretical framework of inertial-navigation-based continuous positioning. In this context, this review presents a simulation analysis of wellbore trajectory error propagation. The analysis indicates that effective motion constraints and suitable algorithms must be integrated to mitigate error accumulation and environmental noise. Nevertheless, current theoretical methods and engineering conditions are not yet sufficient to fully overcome the existing technical challenges. Looking ahead, the integration of multisensor measurement and artificial intelligence holds considerable potential for advancing continuous wellbore trajectory measurement.

Overall, the existing literature underscores the importance of wellbore attitude and trajectory measurement in subsurface hydrocarbon exploration. With coordinated advances in quantum sensing and artificial intelligence, and their integration with intelligent drilling-tool control and decision-making, the theoretical foundations of underground navigation and positioning are expected to gain further significance.

## Figures and Tables

**Table 1:** Typical sensor configuration and attitude accuracy.

Company /Institution	Tool Type	Wellbore Attitude Accuracy		
		Azimuth	Inclination	Tool Face
Schlumberger (Houston, TX, USA)	Solid-state Gyro (GyroLink) MEMS Gyro (Gyrosphere)	±0.1°	±0.05°	±0.1°
Baker Hughes (Houston, TX, USA)	Magnetometer (MWD) [7]	±1°	±0.1°	±1°
Halliburton (Houston, TX, USA)	BaseStar (MWD)	±1°	±0.1°	±1°
Scientific Drilling (Houston, TX, USA)	Keeper MWD; URSA MWD Solid-state Gyro	±0.1°	±0.1° (0°~80°)	±0.1°
	Magnetometer (Falcon X)	±0.25°	±0.15°	±0.15°
Weatherford (Houston, TX, USA)	TrendLine Gyro-while-drilling	±1°	±0.1°	±1°
Beihang University (Beijing, China)	FOG (FMWD,FIW) [8]	±1°	±0.1°	±0.1°

Note: Unless otherwise specified, the product performance specifications listed in the table were compiled from product datasheets or technical specifications publicly available on the official websites of Schlumberger, Baker Hughes, Halliburton, Scientific Drilling, and Weatherford. Because the definitions of performance metrics and test conditions may differ among manufacturers, the values are provided only as a summary of representative information.

**Table 2:** Applicability of different types of gyroscopes in oil and gas well engineering.

Parameter	Mechanical Gyro	MEMS Gyro	Ring Laser Gyro	Fiber Optic Gyro
Bias stability (°/h)	Strategic Grade (<0.001) Navigation Grade (0.001~0.05)	Tactical Grade (0.1~5) Consumer Grade (>5)	Strategic Grade (<0.001) Navigation Grade (0.001~0.05)	Strategic Grade (<0.001) Navigation Grade (0.001~0.05) Tactical Grade (0.1~5)
Size adaptability	Conventional wellbore. Incapable of near-bit installation.	Slim hole or conventional wellbore. Capable of near-bit installation.	Challenging to integrate into downhole tools because of its size and power requirements	Slim hole or conventional wellbore. Capable of near-bit installation with flexible design
Working temperature	Warm-up required before operation. Typically up to 100 °C. Fluid-floated gyro (e.g., SDI GyroMWD) can reach 150 °C.	Maximum operating temperature: 150 °C. Significant temperature drift.	Not capable of downhole operation. Maximum temperature: 70 °C	Fast start-up. Maximum operating temperature 85 °C. Applicable for vertical build-up section or wells within 2000 m. reach 175 °C with a thermal flask.
Vibration shock	Some mechanical rotor gyros are relatively sensitive to vibration	It has good vibration resistance but a high noise level.	Poor vibration resistance makes it unsuitable for drilling environment.	Fully solid-state packaging. Excellent vibration resistance.
Power consumption	Precision rotor gyros can reach tens of watts. Certain models can meet downhole operation requirements (e.g., Keeper Gyro)	As low as mW level, with total system under 2 W.	Power consumption is tens of watts or more, which does not meet downhole power requirements.	The total power consumption of the three-axis gyro does not exceed 10 W, meeting downhole power requirements.

Note: This table summarizes the main characteristics and downhole applicability of different gyroscope types based on the cited literature and technical sources.

**Table 3:** Research framework for gyro-based wellbore attitude determination.

Research Object	Research Aim	Expected Effect
Inertial sensor	Error characterization; Inertial sensor denoising.	Reduce the amplitude of gyroscope/accelerometer noise, ensuring stability of attitude angle measurement
Working environment	Construct a physical model for error compensation under drilling conditions, enabling highly reliable measurement under dynamic working conditions	High-precision measurement of wellbore attitude angle during continuous drilling or in dynamic environments
Measurement scheme	Auxiliary information constraints, compensate for framework errors in the solution	Improve system-level solution performance and wellbore attitude accuracy

**Table 4:** Simulation parameter settings.

Simulation	Parameter
Gyroscope errors	Bias: 0.05°/h; Zero-mean Gaussian white noise: 0.05°/h (1σ)
Accelerometer errors	Bias: 100 μg; Zero-mean Gaussian white noise: 100 μg (1σ)
Altitude angles	Toolface angle: 60°, Azimuth angle: 45°
Latitude and Longitude	Latitude: 40°, Longitude: 116°
Sampling frequency	10 Hz
Dwell time at each attitude point	30 s

**Table 5:** Simulation parameters for 1-h static navigation.

Simulation	Parameter
Gyroscope errors	Bias: 0.05°/h Random noise: 0.05°/h (1σ)
Accelerometer errors	Bias: 100 μg Random noise: 100 μg (1σ)
Altitude angles	Inclination angle: 90° Toolface angle: 0° Azimuth angle: 0°
Latitude and Longitude	Latitude: 40°, Longitude: 116°
Navigation frequency	10 Hz

## Data Availability

Simulation datasets were generated in this study, and the relevant simulation parameters are provided in the manuscript to ensure the reproducibility of the results.

## References

[1] Huo A., Zhang K., Zhang S. (2024). Attitude control of rotary steering drilling stabilized platform based on improved deep deterministic policy gradient. SPE J..

[2] Yang Y., Di Q., Xie Q., Wang Z., Li H., Zhang L. (2024). High temperature directional inclinometer while drilling based on MEMS gyro. Chin. J. Geophys..

[3] Diao B., Gao D., Liu Z., Wu H. (2024). Well depth measured with MWD error correction and calculation of borehole position uncertainty. Pet. Drill. Tech..

[4] McGregor A., Willerth M., Agarwal N. Optimizing Wellbore Trajectories for Closed Loop Geothermal Operations. Proceedings of the SPE Offshore Europe Conference and Exhibition.

[5] Yang J., Yang X., Zhang Y. (2025). Online error compensation of magnetometer while drilling based on magnetic inertial planet optimization. Measurement.

[6] De Oliveira V., Rivas M., Osman A., Moorkan S. Novel Solid-State Gyro While Drilling Technology Provides Highest Positional Accuracy in Horizontal Wells. Proceedings of the International Petroleum Technology Conference.

[7] Dreisig H., Romanus A., Pozo M., Cabrera N., Aghai F., Pubanz S., Hansen C., Anda D., Gjertsen M., Klemme C. Accurate Wellbore Placement with Rotating Continuous Surveys while Saving Time and Cost in Non-Conventional Shale Play. Proceedings of the ADIPEC.

[8] Xu H., Wang L., Zu Y., Gou W., Hu Y. (2023). Application and Development of Fiber Optic Gyroscope Inertial Navigation System in Underground Space. Sensors.

[9] Gu H., Wu Y., Li X., Hou Z. (2025). Research on Wellbore Trajectory Optimization and Drilling Control Based on the TD3 Algorithm. Appl. Sci..

[10] Yu R., Diao B., Gao D. (2023). Calculation for wellbore trajectory measurement error incorporating magnetic azimuth correction. Pet. Drill. Tech..

[11] Yu R., Diao B., Gao D. (2022). A calculation model for improving the relative borehole uncertainty based on the least square method in relief well. J. Pet. Sci. Eng..

[12] Liu X. (2019). Borehole trajectory uncertainty and its characterization. Pet. Explor. Dev..

[13] Engelsman D., Klein I. (2023). Information-aided inertial navigation: A review. IEEE Trans. Instrum. Meas..

[14] Saasen A., Poedjono B., Anesbug G., Zachman N. (2021). Efficient removal of magnetic contamination from drilling fluids: The effect on directional drilling. J. Energy Resour. Technol..

[15] Liu Y., Li X., Zhang X., Feng Y. (2014). Novel Calibration Algorithm for a Three-Axis Strapdown Magnetometer. Sensors.

[16] Yu H., Guo Y., Ye L., Han T., Su S. (2024). Experimental design for the inclination-based linear magnetometers calibration method. IEEE Trans. Instrum. Meas..

[17] Fagaly R., Paulson D., Wikswo J. (2024). Magnetometer Calibration Methods. IEEE Trans. Appl. Supercond..

[18] Batista D., Granziera F., Tosin M., Melo L. (2023). Attitude-Independent Magnetometer Calibration Using Nonlinear Least Squares. IEEE Sens. J..

[19] Guan J., Chen Z., Jiang G. (2025). Research on Calibration Method of Triaxial Magnetometer Based on Improved PSO-Ellipsoid Fitting Algorithm. Electronics.

[20] Li R., Miao H., Hao Y., Sui Y. (2024). Improved Ellipsoid Fitting Method Based on Tensor Mix Source Separation for Correcting the Three-Component Magnetometer. IEEE Sens. J..

[21] Shi L., Liu Y. (2022). Three-step autonomous calibration method for low-cost MEMS inertial/magnetic sensors. IEEE Trans. Magn..

[22] Yu H., Ye L., Guo Y., Su S. (2020). An effective in-field calibration method for triaxial magnetometers based on local magnetic inclination. IEEE Trans. Instrum. Meas..

[23] Malekizadeh A., Afarideh H., Mohamadian M., Esmaeili Sani V. (2024). Calibration of accelerometer and magnetometer sensors of MWD systems in directional drilling application. Meas. Sci. Technol..

[24] Ji S., Zhang C., Lin T., Gao S., Lian A. (2024). A bi-vector calibrated algorithm of fiber optic gyroscopes aided magnetometers based on inclinometer. Meas. Sci. Technol..

[25] Kabirzadeh H., Rangelova E., Lee G., Jeong J., Woo I., Zhang Y., Kim J. (2018). Dynamic Error Analysis of Measurement While Drilling Using Variable Geomagnetic In-Field Referencing. SPE J..

[26] Bulychenkov K. (2023). Overcoming fundamental restrictions: Novel multistation measurement while drilling survey correction. SPE J..

[27] Anda D.E.H., Romanus A., Hansen C., Gjertsen M., Vogt A. Multi-Station Analysis for Advanced Quality Control and Corrections of Rotary Continuous Survey While Drilling. Proceedings of the IADC/SPE International Drilling Conference and Exhibition.

[28] Wang W., Geng Y., Wang K., Si J., Fiaux J. (2018). Dynamic Toolface Estimation for Rotary Steerable Drilling System. Sensors.

[29] Yang H., Rao Y., Li L., Liang H., Luo T., Luo B. (2020). Dynamic measurement of well inclination based on UKF and correlation extraction. IEEE Sens. J..

[30] Li Z., Geng Y.F., Yang Y.S., Wang W.L., Huang S. (2024). Estimation of geomagnetic components under unknown interferences for drilling tools. Meas. Sci. Technol..

[31] Xue Q., Leung H., Wang R., Liu B., Wu Y. (2016). Continuous Real-Time Measurement of Drilling Trajectory With New State-Space Models of Kalman Filter. IEEE Trans. Instrum. Meas..

[32] Liu J., Liu X., Wei L., Peng W., Hu S. (2025). A Multi-Stage Data-Driven Process for Magnetic Azimuth Error Compensation in Horizontal Wells Under Complex Magnetic Environments. Processes.

[33] Liu Q., Kong F., Chen X., Li K. (2025). Research on data completion and generation of downhole drilling tool attitude based on Long Short-Term Memory neural networks. J. Pet. Explor. Prod. Technol..

[34] Liu Q., Kong F., Chen X., Wang K., Li K. (2024). Improving the accuracy of dynamic inclination measurement by machine learning. Sci. Rep..

[35] Yang H., Zhang L., Luo T., Liang H., Li L., Rao Y. (2021). Research on Improving Accuracy of MWD Based on Support Vector Classifier and K-Proximity Method. IEEE Sens. J..

[36] Zhou Y., Xu Y., Cui Y., Wu Y., Li Y. (2025). Accurate prediction of attitude angles in the measurement while drilling system through backpropagation neural networks. Meas. Sci. Technol..

[37] Yang J., Yin F. (2024). Online Error Compensation of Gyroscope in MWD Based on MGMA. IEEE Sens. J..

[38] Hu L., Wang L., Zu Y., Qing Y., Hu Y. (2025). Drilling Tool Attitude Dynamic Measurement Algorithm Based on Composite Inertial Measurement Unit. Mathematics.

[39] Zhang C., Lin T. (2016). A Long-Term Performance Enhancement Method for FOG-Based Measurement While Drilling. Sensors.

[40] Li B., Lu J., Xiao W., Lin T. (2015). In-field fast calibration of FOG-based MWD IMU for horizontal drilling. Meas. Sci. Technol..

[41] Qi F., Meng Y., Yu Y., Zhu X. (2023). Research on a High-Precision Measurement Algorithm Using FOG-Based Single-Axis RINS for Core Drilling. IEEE Trans. Instrum. Meas..

[42] Wang L., Zhang C., Lin T., Li X., Wang T. (2015). Characterization of a fiber optic gyroscope in a measurement while drilling system with the dynamic Allan variance. Measurement.

[43] Zhang C., Wang L., Gao S., Lin T., Li X. (2017). Vibration Noise Modeling for Measurement While Drilling System Based on FOGs. Sensors.

[44] Wang L., Hu Y., Wang T., Liu B. (2021). Vibration Error Correction for the FOGs-Based Measurement in a Drilling System Using an Extended Kalman Filter. Appl. Sci..

[45] Yang J., Du H., Wang S. (2025). Online Error Parameter Identification of Gyroscope Based on SMO in Drilling. IEEE Sens. J..

[46] Yan L., Shi Z., Li B., Dokhani V., Zhou Z. A Study on North-Finding Methods for an Intelligent Downhole Inclinometer Instrument Based on MEMS Gyroscopes. Proceedings of the Middle East Oil, Gas and Geosciences Show (MEOS GEO).

[47] Ursenbach K., Mintchev M. Effect of In-Drilling Alignment with General Dynamic Error Model on Azimuth Estimation. Proceedings of the 2019 Big Data, Knowledge and Control Systems Engineering (BdKCSE).

[48] Li R., Massoud A., Georgy J., Iqbal U., Zhao J., Noureldin A. (2012). Augmented Fast Orthogonal Search/Kalman Filtering (FOS/KF) Positioning and Orientation Solution Using Mems-Based Inertial Navigation System (INS) in Drilling Applications. Instrum. Sci. Technol..

[49] Sha X., Zhao J., Li J., Zhang C. On Measurement-While-Drilling based on rotational inertial navigation system. Proceedings of the 2016 IEEE Chinese Guidance, Navigation and Control Conference (CGNCC).

[50] Yang J., Wang S. (2024). MWD gyroscope error compensation based on equivalent reverse rotation. IEEE Sens. J..

[51] Sawaryn S. (2021). A Generalized Solution to the Point-to-Target Problem Using the Minimum Curvature Method. SPE Drill. Compl..

[52] Oloro O., Efenedo G., Ukrakpor F. (2022). Application of models for directional drilling technology. Int. J. Adv. Appl. Sci..

[53] Škrjanc Ž., Vulić M. (2016). Comparison of the directional survey calculation methods applied on real well data. Measurement.

[54] Wilson G. (1968). An Improved Method for Computing Directional Surveys. J. Pet. Technol..

[55] Sawaryn S., Thorogood J. (2005). A compendium of directional calculations based on the minimum curvature method. SPE Drill. Complet..

[56] Liu X. (2009). Planning technique of 3d sidetrack wells for bypassing obstacles. Acta Pet. Sin..

[57] Eren T., Suicmez V. (2020). Directional drilling positioning calculations. J. Nat. Gas Sci. Eng..

[58] Codling J. The Effect of Survey Station Interval on Wellbore Position Accuracy. Proceedings of the SPE Annual Technical Conference and Exhibition.

[59] ElGizawy M., Lowdon R., Edmunds M., Breen M., Winner E. Accuracy Prediction of Zero-Survey Time Definitive Dynamic MWD Surveys. Proceedings of the Abu Dhabi International Petroleum Exhibition & Conference.

[60] Huang W., Wu M., Chen L., She J., Hashimoto H., Kawata S. (2020). Multiobjective drilling trajectory optimization considering parameter uncertainties. IEEE Trans. Syst. Man Cybern. Syst..

[61] Wang X., Cheng R., Zhao G., Zheng W. (2023). Borehole Trajectory Optimization System Based on Snake Optimization Algorithm. Proceedings of the 2023 IEEE 3rd International Conference on Power, Electronics and Computer Applications (ICPECA).

[62] Gao Y., Wang N., Ma Y. (2023). L2-SSA-LSTM prediction model of steering drilling wellbore trajectory. IEEE Access.

[63] Huang M., Zhou K., Wang L., Zhou J. (2024). Application of long short-term memory network for wellbore trajectory prediction. Pet. Sci. Technol..

[64] Xia X., Bhatt N., Khajepour A., Hashemi E. (2023). Integrated Inertial-LiDAR-Based Map Matching Localization for Varying Environments. IEEE Trans. Intell. Veh..

[65] Tong P., Yang X., Yang Y., Liu W., Wu P. (2023). Multi-UAV Collaborative Absolute Vision Positioning and Navigation: A Survey and Discussion. Drones.

[66] Huang J., Hu Z., Yi W. (2025). Complex Environmental Geomagnetic Matching-Assisted Navigation Algorithm Based on Improved Extreme Learning Machine. Sensors.

[67] Noureldin A., Irvine-Halliday D., Mintchev M. (2002). Accuracy limitations of FOG-based continuous measurement-while-drilling surveying instruments for horizontal wells. IEEE Trans. Instrum. Meas..

[68] Jurkov A., Cloutier J., Pecht E., Mintchev M. (2011). Experimental Feasibility of the In-Drilling Alignment Method for Inertial Navigation in Measurement-While-Drilling. IEEE Trans. Instrum. Meas..

[69] Wang L., Wang Y., Deng Y., Noureldin A., Li P. (2020). Drilling trajectory survey technology based on 3D RISS with a single fiber optic gyroscope. Optik.

[70] Ji S., Zhang C., Ran L. (2022). An Attitude Improvement Method of FOG-Based Measurement-While-Drilling Utilizing Backtracking Navigation Algorithm. IEEE Sens. J..

